# A CD36 ectodomain mediates insect pheromone detection via a putative tunnelling mechanism

**DOI:** 10.1038/ncomms11866

**Published:** 2016-06-15

**Authors:** Carolina Gomez-Diaz, Benoîte Bargeton, Liliane Abuin, Natalia Bukar, Jaime H. Reina, Tudor Bartoi, Marion Graf, Huy Ong, Maximilian H. Ulbrich, Jean-Francois Masson, Richard Benton

**Affiliations:** 1 Center for Integrative Genomics, Faculty of Biology and Medicine, University of Lausanne, CH-1015 Lausanne, Switzerland; 2 Centre for Self-Assembled Chemical Structures (CSACS), McGill University, Montreal, Quebec, Canada H3A 2K6; 3 Département de Chimie, Université de Montréal, Montreal, Quebec, Canada H3C 3J7; 4 BIOSS Centre for Biological Signalling Studies, University of Freiburg, 79104 Freiburg, Germany; 5 Faculty of Pharmacy, Université de Montréal, Montreal, Quebec, Canada H3C 3J7; 6 Department of Nephrology, University of Freiburg Medical Center, 79106 Freiburg, Germany

## Abstract

CD36 transmembrane proteins have diverse roles in lipid uptake, cell adhesion and pathogen sensing. Despite numerous *in vitro* studies, how they act in native cellular contexts is poorly understood. A *Drosophila* CD36 homologue, sensory neuron membrane protein 1 (SNMP1), was previously shown to facilitate detection of lipid-derived pheromones by their cognate receptors in olfactory cilia. Here we investigate how SNMP1 functions *in vivo*. Structure–activity dissection demonstrates that SNMP1's ectodomain is essential, but intracellular and transmembrane domains dispensable, for cilia localization and pheromone-evoked responses. SNMP1 can be substituted by mammalian CD36, whose ectodomain can interact with insect pheromones. Homology modelling, using the mammalian LIMP-2 structure as template, reveals a putative tunnel in the SNMP1 ectodomain that is sufficiently large to accommodate pheromone molecules. Amino-acid substitutions predicted to block this tunnel diminish pheromone sensitivity. We propose a model in which SNMP1 funnels hydrophobic pheromones from the extracellular fluid to integral membrane receptors.

## Abstract (web summary)

The CD36-related Sensory Neuron Membrane Protein 1 (SNMP1) facilitates pheromone detection by insect odorant receptors. Here Gomez-Diaz *et al.* show that the SNMP1 ectodomain is essential for function and propose that it forms a tunnel that transports pheromones from the extracellular fluid to their cognate receptors.

## Graphical abstract

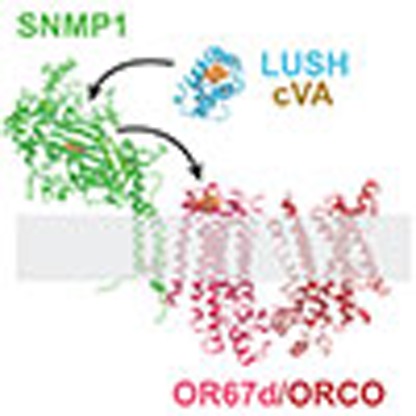

The CD36 (cluster of differentiation 36) family of transmembrane proteins is broadly conserved in animals but displays remarkable functional versatility^1^,^2^,^3^,^4^. The three mammalian CD36 proteins (CD36, SR-BI and LIMP-2) are implicated in lipoprotein scavenging, fatty acid transport, innate immune signalling, cell adhesion, lysosomal protein sorting and gustatory fat detection^1^,^2^,^3^,^4^. Consistently, mutation or misregulation of CD36 proteins in humans has been linked to several diseases, including arterial hypertension, diabetes, cardiomyopathy and epilepsy^1^,^2^,^3^,^4^.

Despite the importance of these proteins, the precise mechanism(s) by which they function is enigmatic. Many molecular studies on mammalian CD36 family members have exploited *in vitro* biochemical assays, which have identified a large number of lipidic and protein ligands (for example, fatty acids, oxidized low-density lipoproteins, thrombospondin 1 and hexarelin) and correspondingly diverse ligand-binding regions in the large ectodomain of these proteins^1^,^2^,^3^,^4^. The downstream consequences of ligand/CD36 protein interactions have mostly been analysed in heterologous cell culture expression systems. Such studies have revealed potential roles for these proteins in mediating ligand translocation across the membrane^5^,^6^, receptor-mediated ligand internalization^7^, transfer of ligands to other cell surface signalling receptors^8^ or direct activation of intracellular signalling cascades (for example, via Lyn and Yes tyrosine kinases^9^). However, demonstration of the relevance of many of these biochemical and cellular properties of CD36 proteins in their native environment has rarely been tested. In part, this reflects the challenge of molecular genetic analysis of CD36 family members that have broad tissue expression and multiple, essential functions.

The genetic model, *Drosophila melanogaster*, offers a powerful system to investigate CD36 protein function *in vivo*. *Drosophila* possesses a repertoire of 14 CD36-like proteins^10^ (Fig. 1a), most of which have tissue-specific expression patterns, suggesting that they have distinct roles^11^. Two family members, NINAD (neither inactivation nor afterpotential D) and Santa Maria, are important for transport of dietary carotenoids from the gut to the photoreceptors^12^,^13^. Others, such as Croquemort and Peste, have been implicated in immune recognition, as they are required for the uptake of *Staphylococcus aureus* and Mycobacteria, respectively, at least in *Drosophila* cell lines^7^,^14^. In flies, Croquemort and another CD36 homologue, Debris Buster, act in phagosome maturation in epidermal cells during clearance of degenerating neural processes^15^.

We, and others, previously characterized a *Drosophila* CD36 family member called sensory neuron membrane protein 1 (SNMP1; originally named SNMP)^16^,^17^,^18^. *Drosophila* SNMP1, as well as its orthologues in other insects, is expressed in olfactory sensory neurons (OSNs) that detect lipid-derived pheromones^19^,^20^,^21^,^22^,^23^. SNMP1 is targeted to the dendritic cilia that are exposed to external chemical signals, where odorant receptors (ORs) are located (Fig. 1b). The best-characterized function of *Drosophila* SNMP1 is in OSNs expressing OR67d that, together with the obligate OR co-receptor ORCO^24^, detects the male sex pheromone (*Z*)-11-octadecenyl acetate (*cis*-vaccenyl acetate, cVA)^25^. Loss of SNMP1 drastically reduces the sensitivity of these neurons to cVA stimulation^16^,^17^,^18^, but does not affect OR67d/ORCO expression or cilia localization. Although these data implicated SNMP1 as a key component of the pheromone signal transduction pathway, its mechanism of action is unknown.

Several non-mutually exclusive models can be envisaged for the function of SNMP1 in pheromone signalling. SNMP1 could bind free pheromone molecules and/or complexes of pheromones with secreted odorant binding proteins (OBPs)^26^ in the extracellular space (Fig. 1b). These interactions could simply concentrate pheromone molecules in the vicinity of pheromone receptors in the cilia membranes or directly facilitate transfer of these ligands to their cognate OR. SNMP1 could also participate in pheromone transduction by coupling to an intracellular signalling cascade, for example, by recruiting proteins to the cilia membranes via its cytosolic tails. Regardless of the exact mechanism, the genetic requirement for SNMP1 in coupling the presence of extracellular lipidic ligands to downstream cellular responses (that is, neuronal firing) was reminiscent of the function of mammalian CD36 in gustatory sensing of fats and immune recognition of pathogenic bacterial lipids and lipoproteins^27^,^28^. These parallels indicate that SNMP1 might be a relevant model for understanding CD36 proteins *in vivo*.

Here we describe a structure–function dissection of SNMP1, using molecular genetic, cellular, biochemical, electrophysiological and homology modelling approaches. Our data demonstrate that the SNMP1 ectodomain is essential for its function, but the intracellular domains are dispensable. We also show that SNMP1 can be substituted by mammalian CD36, whose ectodomain can interact with insect pheromones. In a structural model of the SNMP1 ectodomain, we identify a tunnel, which we suggest may funnel pheromone molecules to their cognate receptors. Our work provides novel insights into insect pheromone transduction and highlight both conserved and divergent molecular mechanisms of CD36 protein function.

## Results

### Deep evolutionary conservation of SNMP1 function

**Figure 1:**
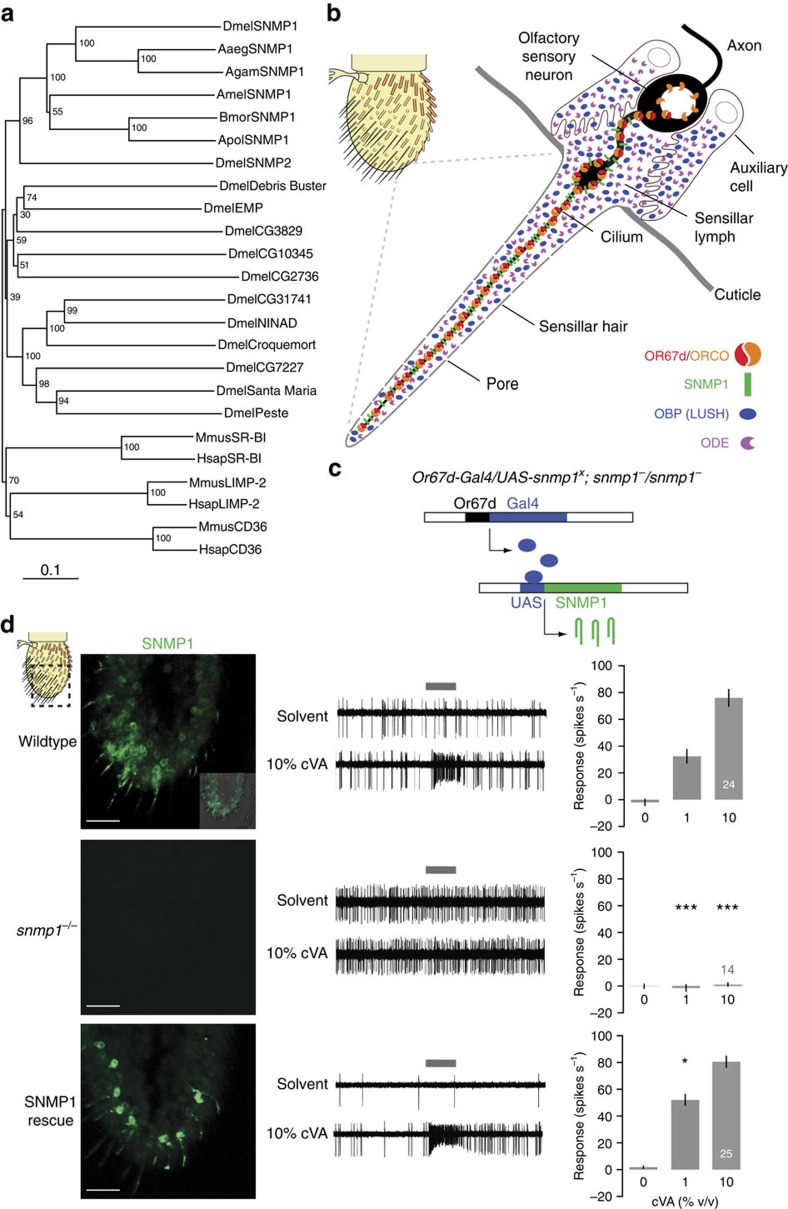
An *in vivo* transgenic system for structure–function dissection of SNMP1. (**a**) Neighbour-joining tree of insect SNMP1 orthologues, and other *Drosophila* and mammalian CD36-related proteins (Dmel, *Drosophila melanogaster*; Aaeg, *Aedes aegypti*; Agam, *Anopheles gambiae*; Amel, *Apis mellifera*; Bmor, *Bombyx mori*; Apol, *Antheraea polyphemus*; Mmus, *Mus musculus*; Hsap, *Homo sapiens*). The tree was built with MEGA5 (ref. 68); bootstrap support (1,000 replicates) is indicated. The scale bar units show amino-acid substitutions per site. (**b**) Schematic representation of the *D. melanogaster* antenna and a detail of a pheromone-sensing trichoid sensillum. The cVA receptor, OR67d, requires an essential co-receptor (ORCO)^16^,^17^. Sensillar lymph contains the OBP LUSH^40^ and odorant-degrading enzymes (ODEs)^69^. (**c**) Schematic representation of the *in vivo* transgenic expression system. The GAL4/UAS system was used to drive the expression of wild-type or mutant versions of SNMP1 (SNMP1^X^), or other CD36-related proteins, in OR67d-expressing neurons in an *snmp1* null mutant background. (**d**) Analysis of wild-type (*w*^*1118*^), *snmp1* homozygous mutant (*snmp1*^*1*^*/snmp1*^*2*^) and SNMP1 rescue (*Or67d-GAL4/UAS-snmp1;snmp1*^*1*^*/snmp1*^*2*^) flies. Left: immunostaining with α-SNMP1 on antennal cryosections. The approximate area shown is indicated with a dashed black square in the antennal cartoon at the top left. An inset in the wild-type immunostaining illustrates the morphological landmarks by the overlay of fluorescence and bright-field images. Scale bars, 20 μm. Centre: representative traces of electrophysiological recordings of OR67d neurons in male flies stimulated with solvent (paraffin oil) or 10% cVA. The grey bar indicates the stimulus time (1 s) in this and all subsequent figures. Right: mean neuronal responses±s.e.m. to the indicated stimuli (see also Supplementary Table 1). As *n* is equal for all concentrations tested for each genotype, it is shown in white or grey within or above only the 10% cVA stimulus bar, in this and all subsequent figures. There are significant statistical differences in neuronal responses due to genotype for both 1 and 10% cVA (Kruskal–Wallis, *P*<0.0001). Significant differences in cVA responses for the different genotypes compared with wild-type (*w*^*1118*^) are indicated in the figure; *P<0.05, ***P<0.001.

To investigate how SNMP1 acts in pheromone detection, we established a pipeline for *in vivo* structure–function analysis. Constructs encoding wild-type or mutant SNMP1—or other CD36-related proteins—were integrated at a common genomic location using phiC31 integrase-based transgenesis^29^. Expression of these transgenes was induced in OR67d neurons using the GAL4/UAS system in an *snmp1* mutant background, which lacks endogenous SNMP1 (ref. 16; Fig. 1c). We assessed both the localization of these proteins to sensory cilia by immunohistochemistry, and their ability to restore neuronal responses to cVA—delivered to the antenna in an airstream—by single sensilla electrophysiological recordings^30^. In this system, transgenically expressed wild-type *D. melanogaster* SNMP1 localizes efficiently to sensory cilia and rescues cVA-evoked responses (Fig. 1d and Supplementary Table 1).

**Figure 2:**
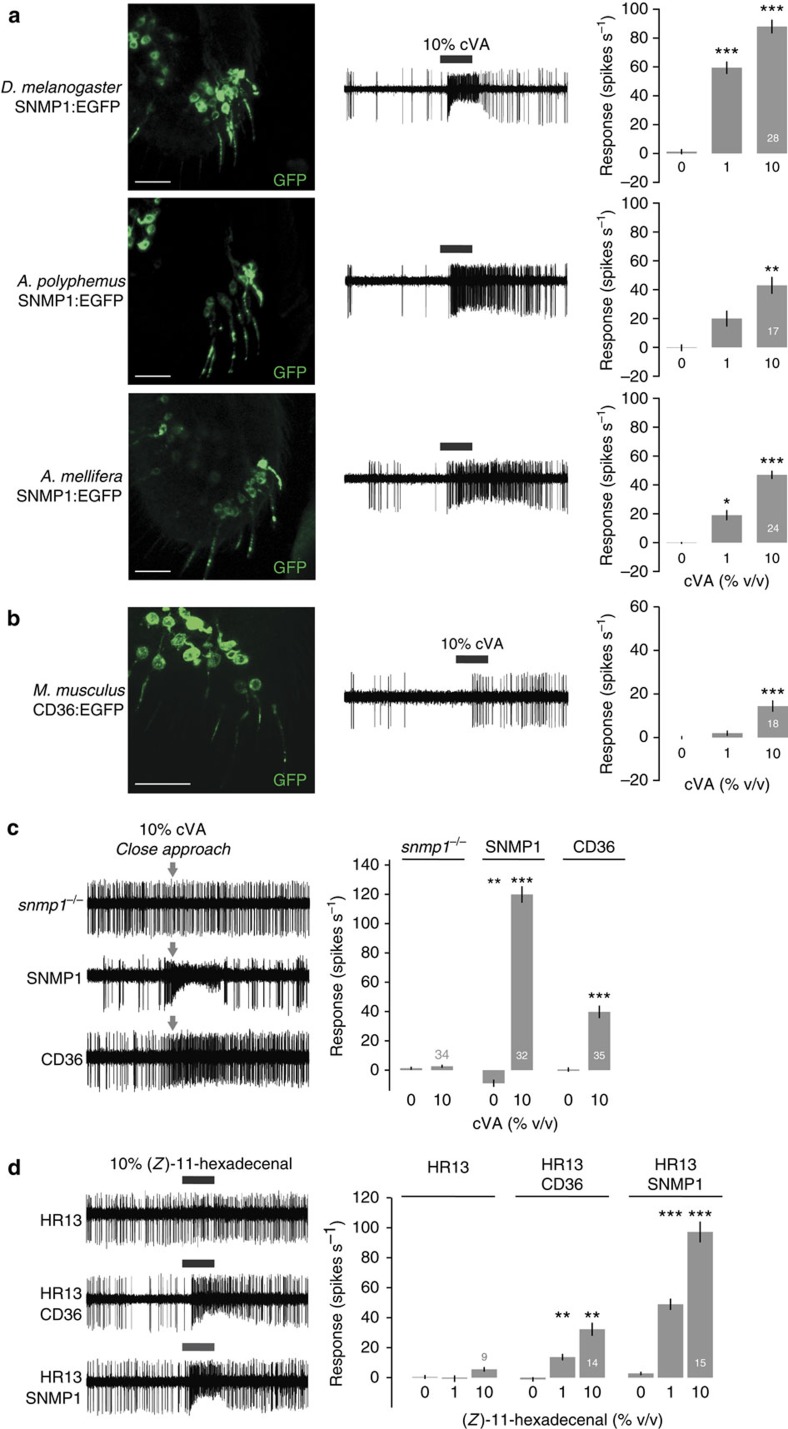
Evolutionary conservation of SNMP1 function. (**a**) Analysis of *snmp1* rescue flies (*Or67d-GAL4/UAS-XXX;snmp1*^*1*^*/snmp1*^*2*^, in this and all equivalent rescue experiments) expressing C-terminal EGFP fusions of SNMP1 from *D. melanogaster*, *A. polyphemus* and *A. mellifera*. Left: immunostaining with α-GFP on antennal cryosections. Scale bars, 20 μm. Centre: representative traces of electrophysiological recordings of OR67d neurons in male flies stimulated with 10% cVA. Right: mean neuronal responses±s.e.m. to the indicated stimuli in each genotype. There are significant statistical differences in neuronal responses to cVA due to genotype (Kruskal–Wallis, *P*<0.0001). Significant differences in cVA responses compared with *snmp1*^*−/−*^ (Fig. 1d) are indicated in the figure; **P<0.01, ***P<0.001. (**b**) Analysis of *snmp1* rescue flies expressing *M. musculus* CD36:EGFP (for immunohistochemistry) or untagged CD36 (for electrophysiology). Left: immunostaining with α-GFP on antennal cryosections. Scale bar, 20 μm. Centre: representative trace of electrophysiological recordings of OR67d neurons in male flies stimulated with 10% cVA. Right: mean neuronal responses±s.e.m. to the indicated stimuli. There is a significant increase in 10% cVA sensitivity in *M. musculus* CD36 rescue when compared with *snmp1*^*−/−*^ (Mann–Whitney test; ***P<0.001). (**c**) Left: representative traces of electrophysiological recordings of OR67d neurons in a close-range stimulation assay with 10% cVA in the indicated genotypes. The grey arrows indicate the approximate time of closest stimulus position to the sensillum. Right: mean neuronal responses±s.e.m. in each genotype. There are significant statistical differences in neuronal responses due to genotype (Kruskal–Wallis, *P*<0.0001). Significant differences in cVA responses of the different genotypes compared with *snmp1*^*−/−*^ are indicated in the figure; *P< 0.05, **P<0.01 and ***P<0.001. (**d**) Left: representative traces of electrophysiological recordings of OR67d neurons lacking OR67d and SNMP1, and ectopically expressing the moth pheromone receptor HR13 in the absence or presence of CD36 and SNMP1, stimulated with 10% (*Z*)-11-hexadecenal. Genotypes: ‘HR13', *UAS-HR13;Or67d*^*GAL4*^*,snmp1*^*1*^*/Or67d*^*GAL4*^*,snmp1*^*2*^); ‘HR13+CD36', *UAS-HR13/UAS-CD36;Or67d*^*GAL4*^*,snmp1*^*1*^*/Or67d*^*GAL4*^*,snmp1*^*2*^); and ‘HR13+SNMP1', *UAS-HR13/UAS-SNMP1;Or67d*^*GAL4*^*,snmp1*^*1*^*/Or67d*^*GAL4*^*,snmp1*^*2*^. Right: mean neuronal responses±s.e.m. in each genotype. There are significant statistical differences in neuronal responses to (*Z*)-11-hexadecenal due to genotype (one-way analysis of variance, *P*<0.0001). Significant differences in cVA responses compared with ‘HR13' are indicated in the figure; **P<0.01, ***P<0.001.

To determine whether the function of SNMP1 is evolutionarily conserved, we first expressed enhanced green fluorescent protein (EGFP)-tagged SNMP1 orthologues from the silk moth *Antheraea polyphemus* and the honeybee, *Apis mellifera*^10^,^21^. Both fusion proteins localize to the sensory compartment and restored responses to cVA, albeit more weakly than those of an equivalent *Drosophila* SNMP1:EGFP (Fig. 2a). These observations indicate that the function of SNMP1 in pheromone detection has been conserved since the divergence of these species more than 350 million years ago^31^, and supports the hypothesis that SNMP1 orthologues function with many different insect ORs to detect diverse pheromones^16^,^32^.

We next asked whether a more divergent member of this family, *Mus musculus* CD36, could substitute for SNMP1. Remarkably, CD36 was both targeted to sensory cilia and capable of restoring, albeit only partially, responses to pheromone (Fig. 2b). We confirmed the capacity of CD36 to support cVA-evoked neuronal activity in a close-range stimulation assay, which permits pheromone presentation at higher concentrations than when delivered in an airstream^33^,^34^. While *snmp1* null mutant OR67d neurons show no detectable responses to cVA presented at close range (Fig. 2c), those expressing either SNMP1 or CD36 exhibit clear increases in spiking frequency (Fig. 2c). We previously showed that SNMP1 is also required for the detection of the moth *Heliothis virescens* sex pheromone (*Z*)-11-hexadecenal by its cognate receptor HR13, when ectopically expressed in OR67d neurons^16^. Consistent with its ability to rescue endogenous cVA sensitivity, CD36 was also capable of enhancing responses of HR13 to (*Z*)-11-hexadecenal (Fig. 2d). Although CD36 is, unsurprisingly, less effective than SNMP1 in these rescue assays (Fig. 2d), our results reveal at least partial conservation in the biochemical function of these CD36 family members, whose last common ancestor existed over 700 million years ago^31^.

### The SNMP1 ectodomain is required for pheromone detection

SNMP1 contains two putative transmembrane domains, separated by a ∼420 amino-acid ectodomain, and flanked by short N- and C-terminal tails (Supplementary Fig. 1). We first confirmed the predicted membrane orientation of this protein by expressing SNMP1 bearing a C-terminal EGFP tag in HEK 293 cells, and assessing accessibility of this tag to immunodetection when cell membranes were unpermeabilized or permeabilized by detergent (Supplementary Fig. 2). While endogenous EGFP fluorescence was detected on intracellular and plasma membranes in both conditions, α-GFP stained these cells only when the membrane was permeabilized, consistent with an intracellular location for the SNMP1 C-terminus (Supplementary Fig. 2).

**Figure 3:**
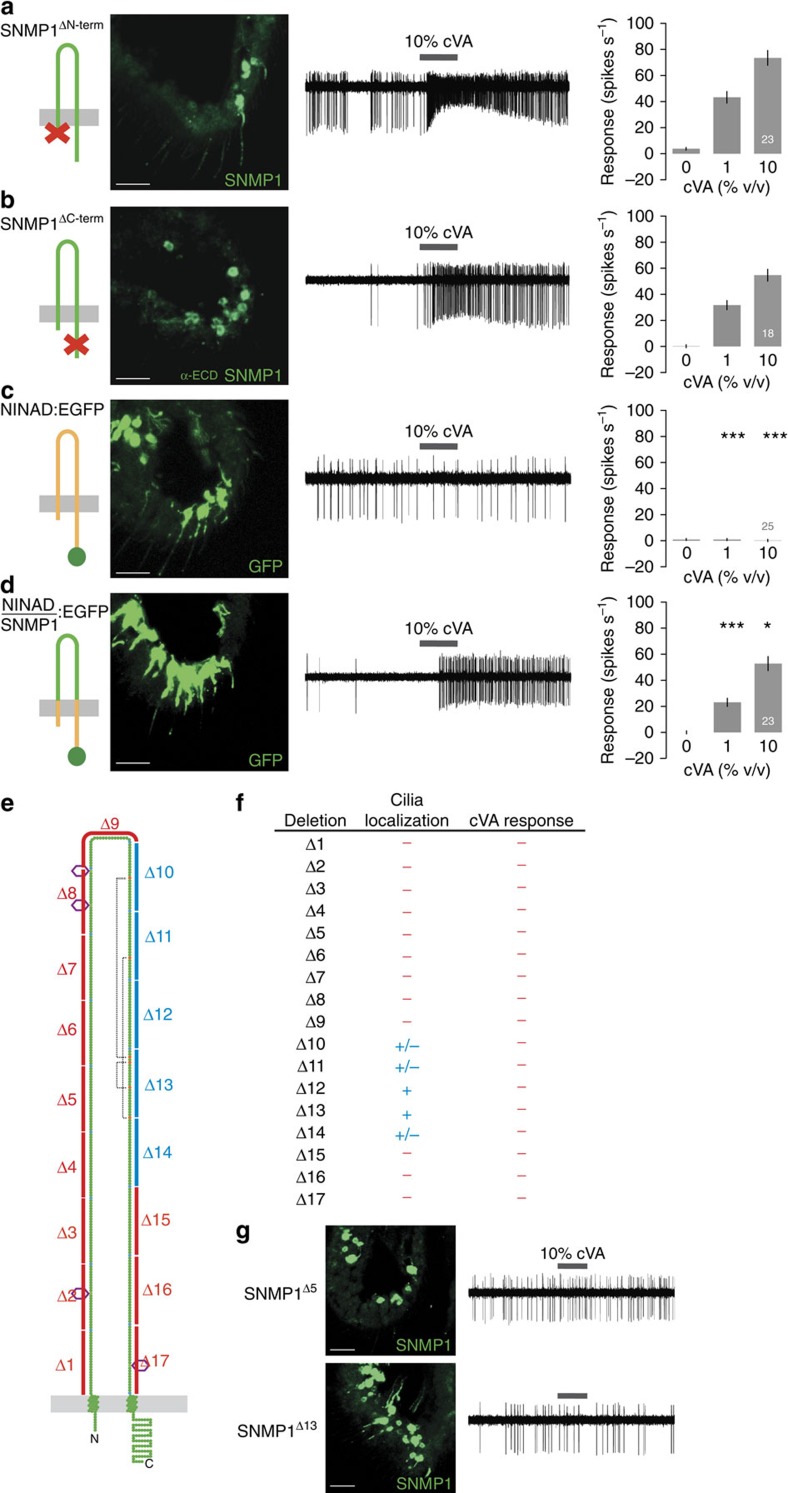
The SNMP1 ectodomain, but not the transmembrane or intracellular domains, is required for pheromone detection. (**a**–**d**) Analysis of SNMP1^ΔN-term^, SNMP1^ΔC-term^, NINAD:EGFP (for immunohistochemistry) or untagged NINAD (for electrophysiology) and SNMP1/NINAD:GFP chimera rescue transgenes. Left: immunostaining with α-SNMP1 (**a**,**b**) or α-GFP (**c**,**d**) on antennal cryosections. Scale bars, 20 μm. As SNMP1^ΔC-term^ lacks the C-terminal peptide epitope of the SNMP1 antibody^16^, we used an antibody raised against an SNMP1 ectodomain peptide; although this antibody recognizes SNMP1 in soma, it does not label cilia-localized SNMP1, even in wild-type flies, precluding direct visualization of SNMP1^ΔC-term^ in this sensory compartment. Centre: representative traces of extracellular electrophysiological recordings of OR67d neurons in male flies stimulated with 10% cVA. Right: mean neuronal responses±s.e.m. in each genotype. There are significant statistical differences in neuronal responses due to genotype for both 1 and 10% cVA (Kruskal–Wallis, *P*<0.0001). Significant differences in cVA responses of the different genotypes compared with full-length SNMP1 rescue (Fig. 1) are indicated in the figure; *P<0.05, ***P<0.001. (**e**) Schematic representation of SNMP1 in which each amino acid is represented by a circle. Putative N-glycosylation sites are indicated with hexagons and disulfide bonds by dashed grey lines. The 17 ectodomain deletions tested are indicated on the structure in blue or red, representing those that do or do not localize to sensory cilia, respectively. (**f**) Summary of the localization and functional properties of the SNMP1 ectodomain deletion mutants. (**g**) Phenotypes of SNMP1^Δ5^ and SNMP1^Δ13^ rescue properties. Left: immunostaining with α-SNMP1 on antennal cryosections. Scale bars, 20 μm. Right: representative traces of electrophysiological recordings of male flies stimulated with 10% cVA.

We next tested the requirement for the cytosolic regions in mediating SNMP1 function by expressing truncated versions of the protein that lack either the N-terminal or C-terminal tails. Both of these deletion variants localize to cilia and are capable of restoring responses to cVA (Fig. 3a,b). To determine whether the transmembrane regions of SNMP1 are critical, we generated a chimeric protein in which these domains (as well as the cytosolic regions) were replaced with those of another *Drosophila* CD36 protein, NINAD^12^,^13^. A non-chimeric NINAD:GFP fusion protein localizes to the sensory compartment (Fig. 3c), but is unable to rescue responses to cVA delivered in an airstream (although NINAD can support very weak cVA-evoked activity in a close-range stimulation assay (Supplementary Fig. 3)). By contrast, the NINAD/SNMP1:GFP chimera, which contains only the ectodomain from SNMP1, restores cVA responsiveness (Fig. 3d). Together, these results indicate that only the specific extracellular region of SNMP1 is essential for pheromone detection, although it is likely that this domain needs to be anchored to the sensory membrane by transmembrane helices.

To identify which regions of the ectodomain are important, we expressed a series of 17 versions of SNMP1 bearing non-overlapping 25 amino-acid deletions along the length of this sequence (Fig. 3e and Supplementary Fig. 1). All deletion-bearing proteins failed to rescue cVA responses in *snmp1* mutants (Fig. 3f), supporting the importance of this domain. For most of these proteins, this lack of function can be ascribed to a complete localization defect (Fig. 3f,g). However, a subset of SNMP1 deletion mutants containing one of five contiguous deletions was still detected within sensory cilia (Fig. 3e–g), implicating this part of the protein either directly in signal transduction or indirectly through correct folding of the ectodomain to support pheromone sensing.

### Ectodomain disulfide bonds are essential for SNMP1 function

**Figure 4:**
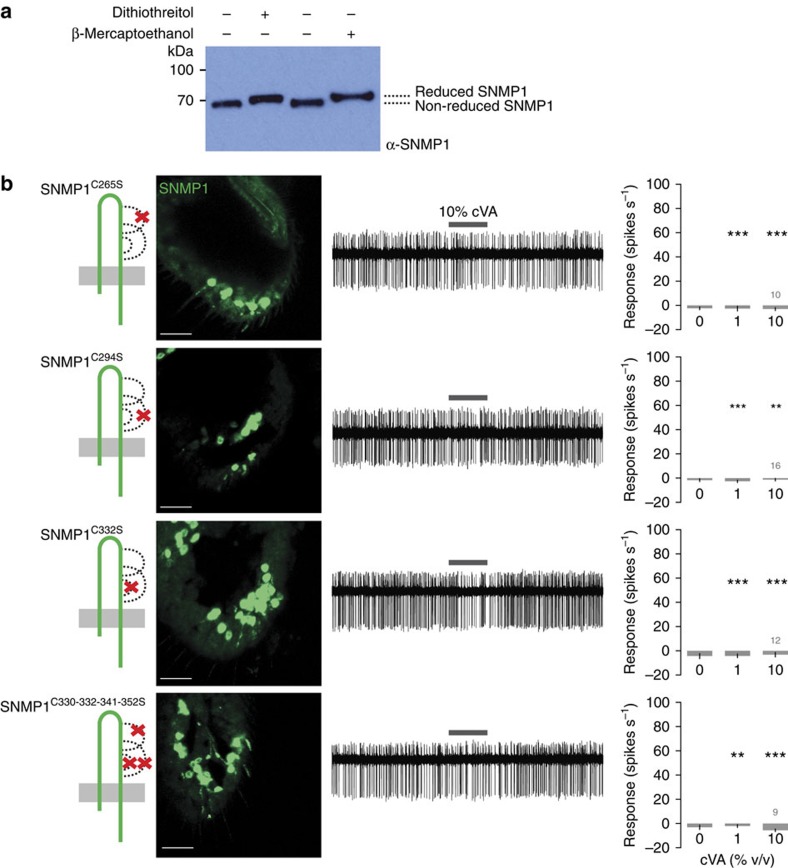
Role of disulfide bridges in SNMP1 trafficking and function. (**a**) Analysis of the presence of intramolecular disulfide bonds in SNMP1 by electrophoretic mobility assay in non-reducing versus reducing conditions. Total antennal protein extracts were resolved by 4–20% SDS–PAGE in the presence or absence of the reducing agents dithiothreitol (100 mM) or β-mercaptoethanol (10%). After western blotting, immunodetection was performed with α-SNMP1. (**b**) Analysis of the rescue properties of SNMP1 mutants lacking one or more disulfide-bond forming cysteine residues as indicated in the cartoons. Left: immunostaining with α-SNMP1 on antennal cryosections. Scale bars, 20 μm. Centre: representative traces of electrophysiological recordings of OR67d neurons in male flies stimulated with 10% cVA. Right: mean neuronal responses±s.e.m. in each genotype. There are significant statistical differences in neuronal responses due to genotype for both 1 and 10% cVA (Kruskal–Wallis, *P*<0.0001). Significant differences in cVA responses of the different genotypes compared with full-length SNMP1 rescue (Fig. 5) are indicated in the figure; **P<0.01, ***P<0.001.

The SNMP1 ectodomain contains six cysteines that are conserved across most CD36 family members (Supplementary Fig. 1). In mammalian CD36, these residues form intramolecular disulfide bonds and have also been implicated in the formation of intermolecular cysteine bridges to permit assembly of multimeric CD36 protein complexes^35^,^36^. To test whether SNMP1 forms intra- and/or intermolecular disulfide bridges we compared the electrophoretic mobility of SNMP1 by SDS–polyacrylamide gel electrophoresis/western blotting of antennal protein extracts under reducing or non-reducing conditions. In the presence of either dithiothreitol or β-mercaptoethanol, SNMP1 migrates slower than in the absence of these reducing agents (Fig. 4a and Supplementary Fig. 4a). These observations are consistent with SNMP1 forming intramolecular disulfide bridges, to impose a more compact conformation of the protein. Under all conditions, we detected only a single band of ∼70 kDa, which is slightly higher than the predicted molecular weight of this protein (∼62 kDa). This result contrasts with detection of higher-molecular-weight species for CD36 under non-reducing conditions^36^, and suggests that SNMP1 does not form covalent intermolecular disulfide bridges.

To confirm the absence of intrinsic multimerization properties of SNMP1, we performed single-molecule imaging of SNMP1:EGFP in a heterologous *Xenopus* oocyte expression system by total internal reflection fluorescence microscopy^37^. This fusion protein was detected in the plasma membrane in bright fluorescent spots of relatively uniform intensity (Supplementary Fig. 5a). EGFP photobleaches within a few seconds under the high-intensity illumination required for single-molecule observation. Examination of the EGFP intensity traces allows determination of the number of separate bleaching steps, and therefore inference of the number of individual EGFP molecules within a spot. We observed that 93% (*n*=1,200) spots displayed a single bleaching step (Supplementary Fig. 5b,c), indicating that SNMP1:EGFP exists principally in monomeric form.

We addressed the contribution of the ectodomain cysteines in SNMP1 function through mutation of individual residues (SNMP1^C265S^, SNMP1^C294S^ and SNMP1^C332S^) predicted to remove one of the three predicted disulfide bridges—based on the pairings established in other CD36 family members^35^—or a mutational combination that removes all three bridges simultaneously (SNMP1^C330-332-341-352S^) (Fig. 4b). All four mutant proteins failed to restore cVA responses, although they could be detected in the sensory cilia, at slightly—or for SNMP1^C332S^, strongly—reduced levels (Fig. 4b). Such phenotypes are consistent with our deletion scanning analysis (Fig. 3e–g), in which SNMP1 deletions covering these cysteine residues (SNMP1^Δ10^-SNMP1^Δ14^) can localize, but not support cVA detection. These results indicate an essential role for disulfide bridges in the signalling, but not targeting, function of the SNMP1 ectodomain.

### Ectodomain glycosylation is required for SNMP1 localization

**Figure 5:**
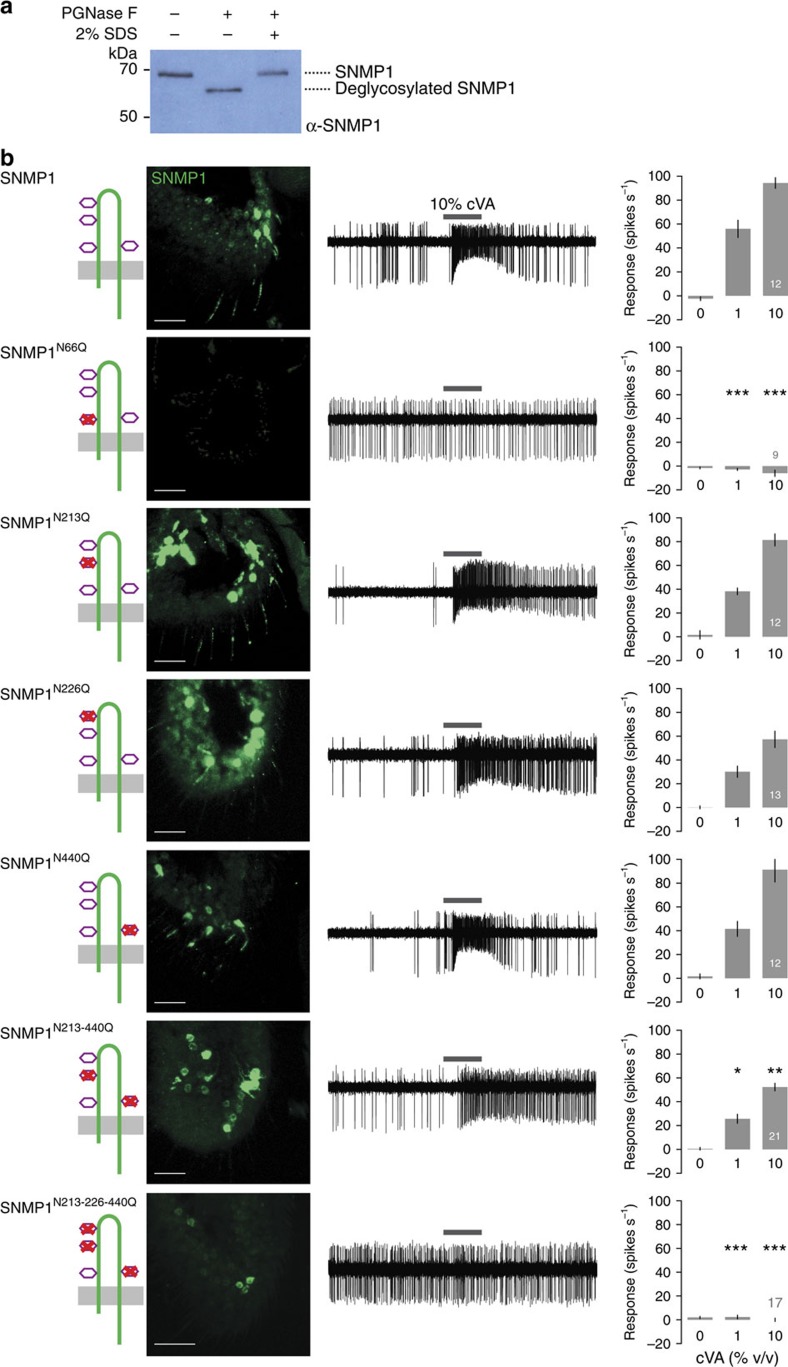
Role of N-glycosylation in SNMP1 trafficking and function. (**a**) Analysis of the N-glycosylation status of SNMP1 by electrophoretic mobility assay. Total antennal protein extracts were incubated with (+) or without (−) PNGase F, in the presence or absence of 2% SDS (to deactivate PNGase F) and then resolved by 10% SDS–PAGE. After western blotting, immunodetection was performed with α-SNMP1. (**b**) Analysis of the rescue properties of SNMP1 mutants lacking one or more putative N-glycosylation sites as indicated in the cartoons. Left: immunostaining with α-SNMP1 on antennal cryosections. Scale bars, 20 μm. Centre: representative traces of electrophysiological recordings of OR67d neurons in male flies stimulated with 10% cVA. Right: mean neuronal responses±s.e.m. in each genotype. There are significant statistical differences in neuronal responses due to genotype for both 1 and 10% cVA (Kruskal–Wallis, *P*<0.0001). Significant differences in cVA responses of the different genotypes compared with full-length SNMP1 rescue are indicated in the figure; *P<0.05, **P<0.01, ***P<0.001.

The ectodomains of mammalian CD36 proteins are characterized by substantial N-glycosylation, which is essential for correct protein folding and/or trafficking^38^. In *Drosophila* SNMP1, we identified four consensus N-glycosylation motifs (NXS/T) at N66, N213, N226 and N440 (Supplementary Fig. 1). Two of these (N66 and N226) are conserved across the majority of CD36 family members, while the others are found only in subsets of SNMP1 orthologues (Supplementary Fig. 1). We tested whether SNMP1 is N-glycosylated *in vivo* by comparing the electrophoretic mobility of endogenous SNMP1 from antennal extracts treated with the N-deglycosylating enzyme PNGase F. While SNMP1 normally runs at ∼70 kDa, PNGase F treatment restores the migration to the predicted molecular weight (∼62 kDa); the effect of PNGase F is abolished by denaturation of this enzyme with SDS (Fig. 5a and Supplementary Fig. 4b). These observations are consistent with SNMP1 bearing sugar modifications at multiple asparagine residues.

We next generated transgenes encoding SNMP1 mutants lacking one or more of these predicted N-glycosylation sites (Fig. 5b). Mutation of the first site, SNMP1^N66Q^, led to complete lack of detectable protein and consequently no restoration of cVA responses (Fig. 5b). By contrast, individual mutation of any of the other sites (SNMP1^N213Q^, SNMP1^N226Q^ or SNMP1^N440Q^) or of two of these together (SNMP1^N213-440Q^) did not produce such drastic phenotypes, although the SNMP1^N226Q^ single mutant and the SNMP1^N213-440Q^ double mutant displayed slightly reduced localization and/or function (Fig. 5b). However, simultaneous mutation of all three of these asparagine residues (SNMP1^N213-226-440Q^) leads to SNMP1 failing to localize to sensory cilia and to restore cVA responses (Fig. 5b). We conclude that N-glycosylation at N66—one of the most conserved glycosylation sites (Supplementary Fig. 1)—fulfils a unique function in protein folding/stability, while sugar modifications of the remaining sites have partially redundant contributions to control trafficking. It is not currently possible to determine whether N-glycosylation also participates in SNMP1's signalling function in cilia.

### Binding of insect pheromones to a CD36 ectodomain

The necessity and sufficiency for the SNMP1 ectodomain suggested that it might interact with pheromones or complexes of pheromones with OBPs that are present in the extracellular lymph bathing OSN cilia. Both possibilities would be compatible with the ability of CD36 family members to bind proteins, lipids and lipoproteins^1^,^2^,^3^,^4^. LUSH is an OBP that binds cVA and is required for high-sensitivity neuronal responses to this pheromone^39^,^40^ (Fig. 1b), although its precise function in signal transduction is unclear^34^. Our extensive attempts to detect binding of LUSH to SNMP1 (in the absence and presence of cVA) by *in vivo* co-immunoprecipitation or cell culture surface-binding assays failed to provide evidence for SNMP1/LUSH complexes. Although these data do not rule out an interaction between these proteins, they suggest that SNMP1 and LUSH do not form a stable complex.

**Figure 6:**
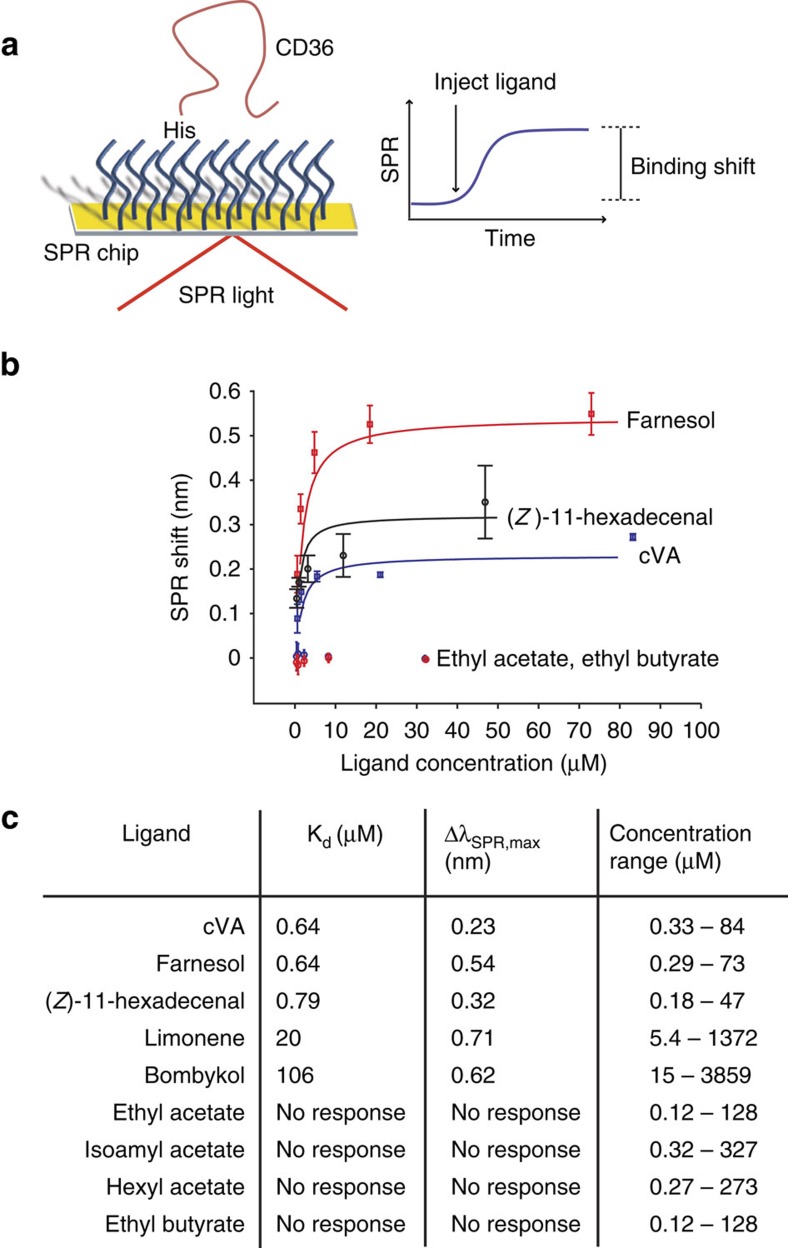
The CD36 ectodomain binds insect pheromones. (**a**) Schematic representation of the SPR method for assessing small-molecule interactions with a CD36 sensor chip (adapted from ref. 70). (**b**) Concentration–response curves of Δ*λ*_SPR_ for the indicated ligands, which were used to calculate corresponding dissociation constants (*K*_d_). (**c**) *K*_d_ and maximum SPR signal (Δ*λ*_SPR,max_) for the indicated compounds.

While LUSH is important for pheromone detection, this class of perireceptor protein is not absolutely essential, as responses of ORs to pheromones have been described in the presence of SNMP1 but without the relevant OBP^16^,^34^,^41^. These observations imply that pheromones alone might be able to bind directly to SNMP1. Unfortunately, we were unable to express the ectodomain of SNMP1 recombinantly in bacterial or insect cell expression systems, precluding our testing of this hypothesis. However, given the conservation in structural features we have found between mammalian CD36 and SNMP1, as well as the ability of mammalian CD36 to substitute for SNMP1 in the detection of both cVA and (*Z*)-11-hexadecenal (Fig. 2b–d), we asked whether pheromone molecules could be bound by this homologous family member using a CD36 sensor chip^42^. In this assay, interaction between the CD36 ectodomain—immobilized on a self-assembled monolayer—and small molecules is measured by surface plasmon resonance (SPR)^42^ (Fig. 6a). We tested three insect pheromones, cVA, (*Z*)-11-hexadecenal and bombykol (a sex pheromone of the moth *Bombyx mori*^26^), as well as two compounds, farnesol and limonene, which are detected by other classes of SNMP1-expressing OSNs^43^,^44^. All five of these exhibited binding as measured by SPR (Fig. 6b,c and Supplementary Fig. 6), with the lowest dissociation constants (representing highest affinity interactions)—for cVA, farnesol and (*Z*)-11-hexadecenal—similar in magnitude to those for known CD36-binding peptides^42^. To examine the specificity of these interactions, we tested four other chemicals that only activate non-SNMP1-expressing neurons (ethyl acetate, isoamyl acetate, hexyl acetate or ethyl butyrate)^33^. None of these showed evidence of interactions (Fig. 6b,c and Supplementary Fig. 6). Together, these results are consistent with the ability of insect pheromones to be directly bound by a CD36 ectodomain.

### A protein homology model reveals a putative pheromone tunnel

**Figure 7:**
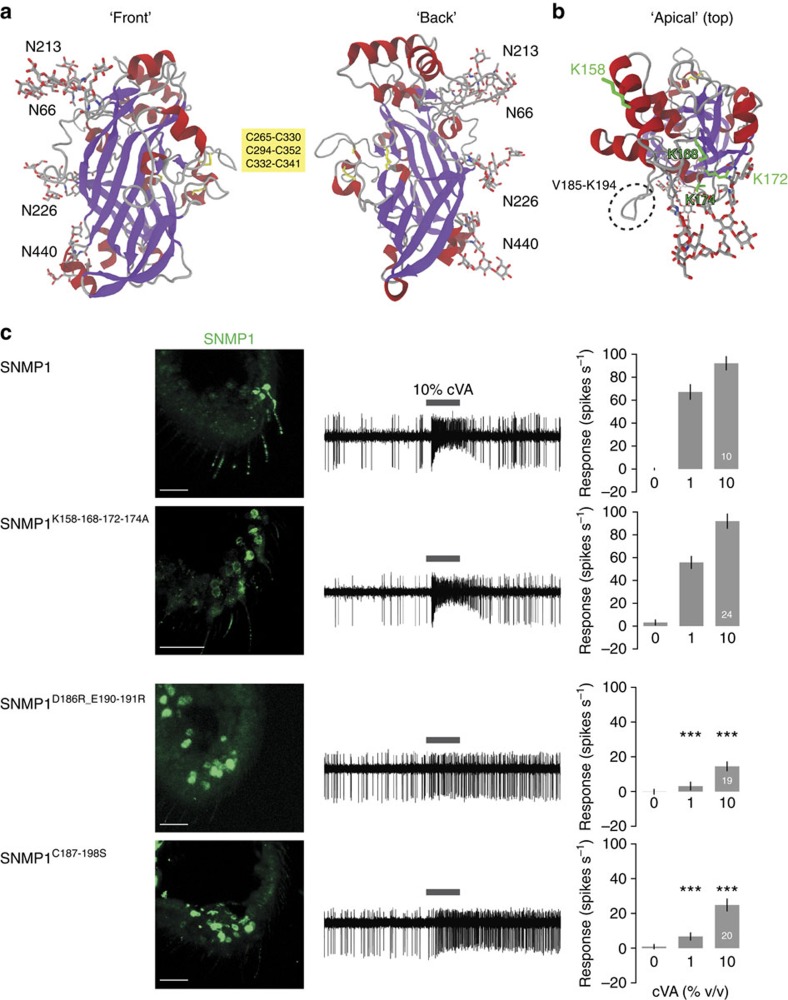
An SNMP1 protein homology model. (**a**) Protein homology model of the SNMP1 ectodomain, from two different views. The α-helices and β-strands are shown in ribbon format in red and purple, respectively. Putative glycosylation sites, cysteine residues and disulfide bonds are indicated in stick format and coloured according to their elements (C: silver, N: blue, O: red, S: yellow). (**b**) ‘Apical' (top) view of the SNMP1 ectodomain. The side chains of K158, K168, K172 and K174 are represented in stick format (green). The SNMP1-specific loop is highlighted with a dashed black circle. (**c**) Analysis of the rescue properties of SNMP1 site-directed mutants lacking the apical basic residues (SNMP1^K158-168-172-174A^) or with substitutions in the SNMP1-specific loop (SNMP1^D186R_E190-191R^ and SNMP1^C187_198S^). Left: immunostaining with α-SNMP1 on antennal cryosections. Scale bars, 20 μm. Centre: representative traces of electrophysiological recordings of OR67d neurons in male flies stimulated with 10% cVA. Right: mean neuronal responses±s.e.m. in each genotype. There are significant statistical differences in neuronal responses due to genotype for both 1 and 10% cVA (Kruskal–Wallis, *P*<0.0001). Significant differences in cVA responses of the different genotypes compared with full-length SNMP1 rescue are indicated in the figure; ***P<0.001.

During the course of our study, X-ray crystal structures of the ectodomain of the mammalian CD36 protein LIMP-2 were obtained^5^,^45^. The amino-acid sequence of the SNMP1 ectodomain is 25.2% identical to that of LIMP-2, and secondary structure predictions for these two proteins are highly comparable (Supplementary Fig. 7). We therefore used homology modelling to build a three-dimensional representation of the SNMP1 ectodomain using the LIMP-2 crystal structure as template (Fig. 7a). The SNMP1 ectodomain model is composed of an antiparallel β-barrel with various short α-helical domains, in particular, a bundle of helices at the apex (Fig. 7a). The four predicted glycosylation sites are located on one face of the ectodomain (Fig. 7a).

The apical region of mammalian CD36 proteins has been implicated in binding of both lipidic and protein ligands. For example, CD36 associates with oxidized phosphatidylcholine and low-density lipoproteins, and mutation of two conserved lysines near the apex (K164 and K166) abrogates these interactions, suggesting the importance of electrostatic interactions with oxidized phospholipid moieties^5^,^46^. These basic residues are not conserved in precise position, but we identified four surface-exposed lysines in this region in *Drosophila* SNMP1 (K158, K168, K172 and K174) (Fig. 7b and Supplementary Fig. 1). However, mutation of these residues to alanine did not affect the function of SNMP1 (Fig. 7c), suggesting that ligands associate with this protein via a different mechanism.

One unique structural element of the SNMP1 apical region is a short loop just after α-helix 6 (V185-K194) (Fig. 7b and Supplementary Fig. 1). This sequence contains several acidic residues, as well as a pair of SNMP1-specific cysteine residues (one lies within the following α-helix). Complete deletion of this loop abolished SNMP1 localization and function (SNMP1^Δ7^, Fig. 3e,f). To test the requirement of this loop more precisely, we charge-reversed the acidic residues (SNMP1^D186R_E190-191R^) and mutated the cysteine pair (SNMP1^C187-198S^). Both of these sets of mutations lead to highly diminished dendritic localization of SNMP1 and a concordant loss in ability to restore cVA responses (Fig. 7c). While these experiments indicate the importance of this loop, we cannot currently distinguish its precise function.

**Figure 8:**
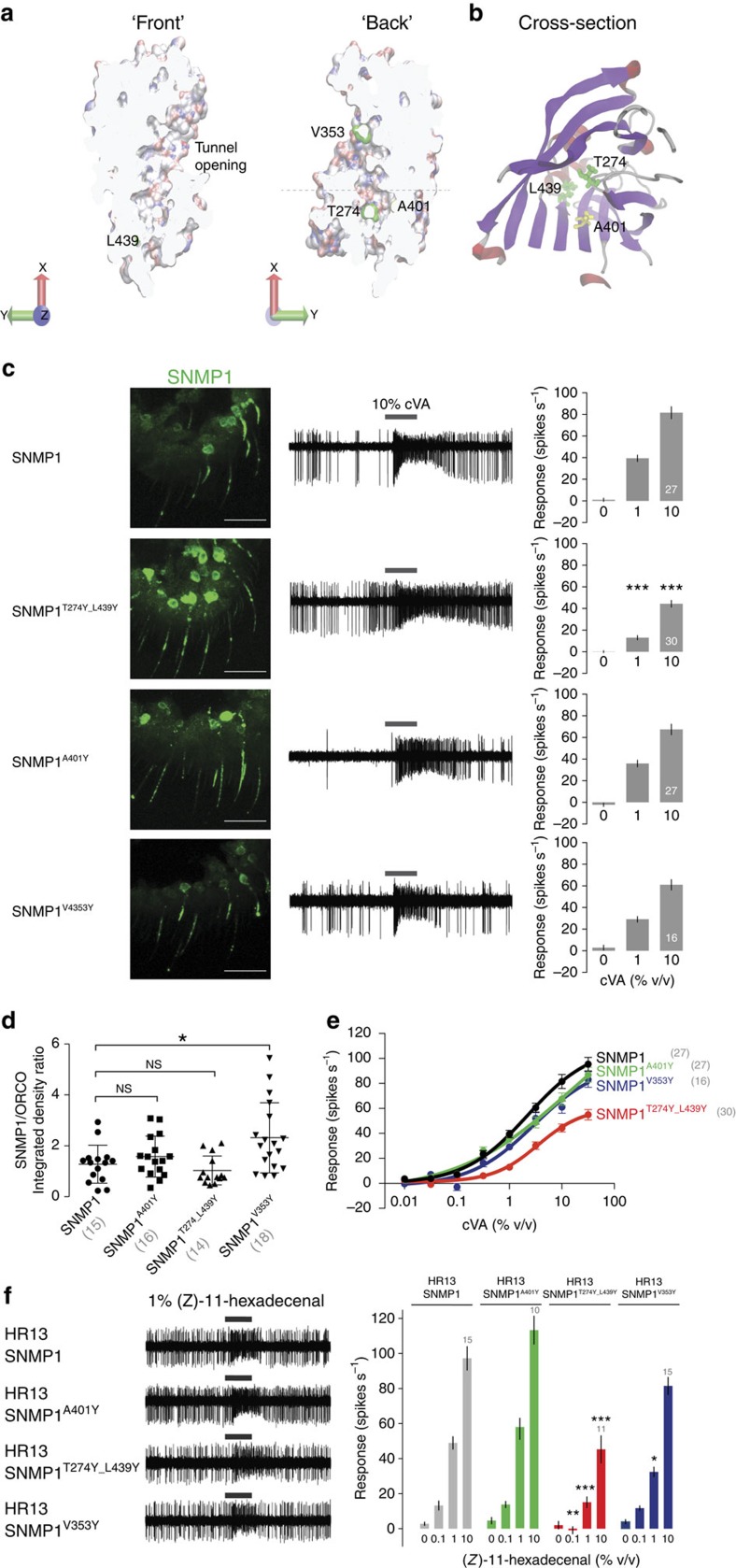
Evidence for a pheromone-conducting tunnel in the SNMP1 ectodomain. (**a**) Representation of the SNMP1 ectodomain tunnel, shown as a slice through the body of the protein. Charged residues on the tunnel surface are coloured (red: negative; white: neutral; blue: positive). Putative tunnel bottleneck residues (T274, L439 and V353, green) and a control tunnel-lining residue (A401, yellow) are highlighted. (**b**) ‘Top' cross-sectional view (at the approximate level of the dashed line in (**a**) of the SNMP1 tunnel, coloured as in Fig. 7a. (**c**) Analysis of the rescue properties of SNMP1 site-directed mutants in which predicted tunnel-lining residues are substituted with larger residues. Left: immunostaining with α-SNMP1 on antennal cryosections. Scale bars, 20 μm. Centre: representative traces of electrophysiological recordings of OR67d neurons (male flies) stimulated with 10% cVA. Right: mean neuronal responses±s.e.m. There are significant statistical differences in neuronal responses due to genotype for 0.1, 1 and 10% cVA (Kruskal–Wallis, *P*<0.001). Significant differences in cVA responses of the different genotypes compared with full-length SNMP1 rescue are indicated; ***P<0.001. (**d**) Analysis of the localization properties of the indicated SNMP1 site-directed mutants using the integrated density ratio of SNMP1 and ORCO immunostaining quantified in OR67d-expressing sensilla. Mean±s.d. in a scatter dot plot is shown. *n* is shown in parenthesis below the labels on the *x* axis. There are significant statistical differences in localization properties due to genotype (Kruskal–Wallis, *P*<0.005). Significant differences of the different genotypes compared with full-length SNMP1 rescue are indicated; *P<0.05). (**e**) Concentration–response curves of OR67d neurons to cVA in the indicated rescue genotypes. Curves were fitted using a log versus response-variable slope model with Prism-GraphPad. Mean responses±s.e.m. are plotted. *n* is shown in parentheses next to the legend labels. (**f**) Left: representative traces of electrophysiological recordings of OR67d neurons lacking OR67d and endogenous SNMP1, and ectopically expressing HR13 in the absence or presence of transgenically expressed SNMP1 proteins, stimulated with 10% (*Z*)-11-hexadecenal. Genotypes: *UAS-HR13/UAS-SNMP1*^*xxxx*^*;Or67d*^*GAL4*^*,snmp1*^*1*^*/Or67d*^*GAL4*^*,snmp1*^*2*^ (‘xxxx': wild type or mutant variant of SNMP1). Right: mean neuronal responses±s.e.m. There are significant statistical differences in neuronal responses to (*Z*)-11-hexadecenal due to genotype (Kruskal–Wallis, *P*<0.0001). Significant differences in cVA responses compared with ‘HR13+SNMP1' are indicated in the figure; *P<0.05, **P<0.01, ***P<0.001.

An unexpected feature of the LIMP-2 crystal structure was an internal tunnel formed predominantly by the β-barrel, which spans most of the length of the ectodomain^5^,^45^. This tunnel was hypothesized to translocate lipidic ligands from the extracellular/extraluminal compartment to the membrane bilayer^5^. We observed the presence of a similar internal cavity in the SNMP1 model (Fig. 8a). This tunnel is sufficiently spacious to accommodate pheromone molecules, such as cVA, and is lined with predominantly neutral amino acids (Fig. 8a), while still retaining the pocket-lining acidic and basic residues that form a network of hydrogen/ionic bonds in the LIMP-2 tunnel (E93, R95, K97, D252, K381 and E413)^5^ (Supplementary Fig. 1). Although direct biochemical demonstration or visualization of movement of pheromone molecules through such a tunnel is technically extremely difficult, we reasoned that blocking this passageway should reduce the ability of SNMP1 to transduce cVA signals. Using tunnel predictions^47^, we identified putative ‘bottleneck residues' within the SNMP1 tunnel (see Methods). After excluding prolines—which might have particular important roles in protein conformation—as well as amino acids with already-large side groups, we identified a pair of residues, T274 and L439, located at a constriction point at the tunnel opening furthest from the apical region (Fig. 8a,b). We mutated both of these residues to tyrosine, which has a bigger side chain. This SNMP1^T274Y,L439Y^ protein localizes indistinguishably from wild-type SNMP1 (Fig. 8c,d), but displays substantial diminishment in its capacity to rescue pheromone responses (Fig. 8c,e). By contrast, replacement with tyrosine of a small residue located in a predicted wider part of the tunnel (SNMP1^A401Y^), affected neither SNMP1 localization (Fig. 8c,d) nor function (Fig. 8c,e). We also attempted to block the entrance of the tunnel by mutation of V353 to tyrosine (Fig. 8a), but this change had little effect on the protein's localization or ability to restore cVA responses (Fig. 8c–e). Finally, we extended functional analysis of these SNMP1 tunnel mutants by analysing their ability to support (*Z*)-11-hexadecenal-evoked responses of HR13 when this receptor is ectopically expressed in OR67d neurons (Fig. 8f). While SNMP1^A401Y^ was indistinguishable from wild type, the bottleneck mutants SNMP1^T274Y,L439Y^ and SNMP1^V353Y^ exhibited either strongly or slightly diminished pheromone sensitivity (Fig. 8f).

## Discussion

SNMP1 was shown to be expressed in pheromone-sensing neurons nearly two decades ago^21^ and genetically implicated in pheromone transduction 10 years later^16^,^17^, but understanding how this CD36 protein acts has been largely elusive. We consider here evidence both for and against potential mechanisms of action of SNMP1 to propose a new model for its function in pheromone signalling.

One model suggested that SNMP1 acts as an inhibitory subunit of OR/ORCO complexes, whose influence is released in the presence of pheromone, thereby leading to an increase in neuronal firing^17^. This proposition was based on the prominent elevated firing of OR67d neurons in the apparent absence of cVA stimulation, which we and others previously interpreted as spontaneous activity^16^,^17^. Recent evidence^18^, however, indicates that this elevated firing reflects instead a highly prolonged, low-level, ligand-evoked activity due to the exposure of flies to environmental sources of cVA (for example, male flies in culture tubes), indicating a role for SNMP1 in controlling pheromone signalling kinetics (see below) rather than inhibiting ORs. Moreover, a purely inhibitory function of SNMP1 is incompatible with observations that expression of SNMP1 is required to enhance sensitivity of responses of ORs to pheromone when expressed in ectopic cells^16^,^23^.

A second model is that SNMP1 transduces signals across the cell membrane to regulate intracellular signalling cascades. Although this mechanism is a prominent feature of mammalian CD36 (refs 7, 48), our demonstration that neither cytosolic tail is required for SNMP1 localization or function argues that SNMP1 is not likely to transmit signals intracellularly, at least for its role in pheromone signalling we have assayed. These observations also imply the existence of a novel type of cilia-targeting signal in SNMP1, as all cilia localization signals identified in other membrane proteins are found in cytoplasmic domains^49^. Moreover, the lack of distinguishing primary structural features of the SNMP1 transmembrane helices compared with other CD36 proteins (Supplementary Fig. 1) and the exchangeability of these sequences by those of NINAD suggest that these regions simply fulfil a structural role in membrane anchoring, rather than contributing a specific function in pheromone transduction. Finally, SNMP1's inability to assemble into homomeric complexes suggests it is unlikely to form, for example, membrane-spanning channels in cilia or rely on receptor clustering for its function.

A third model posits that SNMP1 forms an extracellular platform for capture of pheromones or pheromone/OBP complexes near the sensory cilia membrane^16^,^17^. The sensitivity of the entire ectodomain to small deletions underlies its importance in SNMP1 function. Moreover, although a subregion of the ectodomain encompassing the disulfide bonds appears at least partially dispensable for cilia localization, the complete loss of signalling function of all deletion mutants suggests that this ectodomain acts as a single structural entity, rather than functionally separable subdomains. In support of this model, we have shown that the ectodomain of mammalian CD36, which can partially substitute for SNMP1 *in vivo*, is able to interact with a variety of insect pheromones. Although it is not yet possible to assess direct interactions between *Drosophila* SNMP1 and pheromones, pioneering studies in *A. polyphemus* using a radiolabelled photoaffinity pheromone analogue identified a single labelled ∼70-kDa, antennal-specific membrane protein^50^. Subsequent biochemical purification from olfactory cilia of a protein with these properties led to the initial identification of SNMP1 (ref. 21), suggesting that it is a prominent pheromone-interacting membrane protein in olfactory cilia.

Although SNMP1 is very important for cVA detection, pheromones can directly induce OR-dependent responses in heterologous neurons or other cells, at least when applied at high concentration^33^,^51^. These data imply that pheromones must ultimately interact with ORs, and that SNMP1 is not an integral part of the molecular machinery required for OSN firing. In the context of the third model, what, then, is the role of the SNMP1 ectodomain? The sequence of this region lacks obvious homology to other proteins. Consistently, the three-dimensional structure of the LIMP-2 ectodomain exhibits a novel global protein fold^5^,^45^. The presence of a central cavity in LIMP-2—also preserved in homology models of CD36, SR-BI (ref. 5) and SNMP1—provides an intriguing new hypothesis for the ectodomain in acting as a tunnel for transport of small molecules from the extracellular/extraluminal space to or into the membrane. While direct visualization of movement of molecules through such a tunnel awaits development of appropriate assays, steric blockage of the predicted tunnel in SR-BI by pharmacological or genetic manipulations decreases (by about twofold) cholesterol uptake in cultured cells^5^,^52^. Similarly, we find introduction of larger amino-acid side chains within the presumed SNMP1 tunnel diminishes pheromone sensitivity.

**Figure 9:**
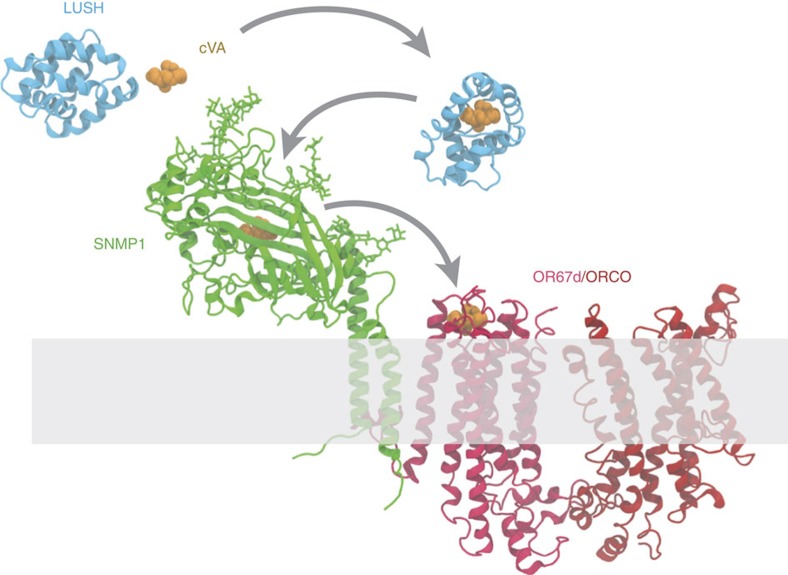
Model for cVA detection. A pheromone molecule is first encapsulated by the OBP LUSH in the extracellular lymph. Direct or indirect interaction of cVA/LUSH with SNMP1 leads to release of the pheromone molecule and its transfer via the ectodomain tunnel in SNMP1 to the ligand-binding site within the OR67d/ORCO complex. *Apo* and cVA-bound LUSH structures are from X-ray crystals (PBD 1T14 (ref. 71) and PDB 2GTE (ref. 39), respectively). The SNMP1 model is the protein homology model shown in Fig. 8, in which N- and C-terminal sequences encoding the transmembrane helices have been added by *de novo* protein folding. The OR67d/ORCO complex is represented as an arbitrarily arranged dimer of a generic OR protein model (OR85b (model 140_12), ORCO (model 310_2)) generated by *de novo* protein folding based on co-evolutionary couplings^53^; the stoichiometry and arrangement of this complex *in vivo* is unknown. The cilia membrane is represented by a transparent grey rectangle.

Together these data lead us to propose a model in which SNMP1 acts by transporting pheromone ligands in the extracellular lymph via an ectodomain tunnel to the cognate pheromone detecting OR in the cilia membrane (Fig. 9). Why should pheromone sensing require such a mechanism? After entering the lymph, pheromones are thought to be encapsulated by OBPs (such as LUSH for cVA^39^,^40^), which induces a conformational change in these proteins^26^,^39^. Subsequent release of pheromone molecules might therefore require energetic input to reverse this conformational change. We hypothesize that pheromone release is triggered by transient interaction of OBP/pheromone complexes with SNMP1. Pheromone molecules must ultimately end up in the ligand-binding pocket of a cognate OR. Although very little is known about the biochemistry of OR/ligand interactions, available data suggest that the binding site lies within the transmembrane regions^53^. The tunnel of SNMP1 might therefore facilitate direct delivery of hydrophobic pheromone molecules to this pocket, thereby protecting them from exposure to the aqueous lymph fluid or odorant degrading enzymes that are abundant in this compartment. Alternatively, and akin to the lipid transport function of CD36 and SR-BI (ref. 54), the tunnel might direct pheromone molecules into the lipid bilayer, from where they move laterally into the OR ligand-binding pocket. This latter possibility would be analogous to the mechanism by which ligands enter the binding site in the free fatty acid receptor GPR40 (ref. 55). Recent analysis of the kinetics of the low-frequency pheromone-evoked responses to high stimulus concentrations in the absence of SNMP1 indicated that this protein is important for both rapid activation and termination^18^. It is possible, therefore, that SNMP1 serves to funnel pheromone molecules both to and from the OR ligand-binding pocket.

Experimental testing of this model is technically challenging, as it demands the functional reconstitution of three transmembrane proteins (SNMP1, OR67d and ORCO), a secreted protein (LUSH) and a radioactively or fluorescently labelled pheromone ligand in an assay system that permits biochemical assessment of dynamic interactions between these components. Moreover, the inability to recapitulate high-sensitivity responses to cVA by misexpression of OR67d, SNMP1 and LUSH in non-pheromone sensing olfactory sensilla, also hints that other signalling components are involved^39^. Nevertheless, the available data do support the insect pheromone detection system as an elegant signalling mechanism that couples low-specificity/high-sensitivity components (such as OBPs and SNMP1 (refs 16, 23, 26)), with high-specificity/low-sensitivity components (that is, pheromone-detecting ORs, which typically recognize a single ligand^33^). This mechanism might underlie the widely documented detection of these important intraspecific signals with both high sensitivity and specificity^26^.

CD36-related genes have been identified across animals, as well as in unicellular eukaryotes^56^, indicating an ancient origin of this superfamily. How functionally distinct CD36 proteins have evolved is poorly understood. Comparison of the properties of SNMP1 with long-studied mammalian homologues provides initial insight into this question. Most strikingly, our observation that mammalian CD36 can compensate for loss of SNMP1 implies the existence of a conserved, ancestral mechanism of action across this functionally diverse protein family.

Unexpectedly, another insect CD36 protein, NINAD, is much less effective in replacing SNMP1 function than the mammalian homologue. We suggest that this reflects a distinction in the evolution of the CD36 repertoires in mammals and insects. While mice and humans have only three, broadly expressed CD36 family members, insect species have evolved a dozen or more proteins^10^,^32^. In *Drosophila*, at least, individual genes exhibit distinct tissue-specific expression patterns^11^. These expression properties might reflect their different roles in, for example, the digestive, sensory or immune system, where they recognize different ligands and might couple to other types of transmembrane receptors. We suggest that, in parallel with their acquisition of unique expression patterns, the insect proteins have become structurally and functionally specialized and hence are unable to effectively substitute for each other. For example, SNMP1's lack of dependence on intracellular domains might reflect its exclusive requirement to transfer pheromone molecules from the lymph to the ORs, without transducing signals intracellularly. By contrast, mammalian CD36 appears to have retained functional versatility, reflecting its implication in multiple distinct signalling roles in different tissues^1^,^2^,^3^,^4^. Future study of chimeric versions of SNMP1 and other insect CD36 proteins might help uncover the conserved and divergent molecular mechanisms by which this protein family recognizes and transduces external signals *in vivo*.

## Methods

### Sequence alignment and phylogenetic analysis

Amino-acid sequences of insect and non-insect CD36-related proteins (Supplementary Fig. 1) were aligned with MUSCLE^57^, using default alignment parameters. This alignment was used to build a Neighbor Joining tree using MEGA 5.2.2 (ref. 58), with the following settings: 1,000 bootstrap replicates, p-distance and gaps handled by pairwise deletion.

### *Drosophila* strains

*Drosophila* stocks were maintained on conventional food medium under a 12-h light:12-h dark cycle at 25 °C. The wild-type genotype used was *w*^*1118*^. Other lines used were *snmp1*^*1*^ and *snmp1*^*2*^ (ref. 16), *Or67d-GAL4* (ref. 59), *UAS-HR13* and *Or67d*^*GAL4*^ (ref. 41). New transgenic lines were generated by standard procedures with the phiC31-based integration system^29^ using the attP40-landing site by Genetic Services Inc. (Cambridge, MA, USA). For all experiments, 4- to 10-day-old flies were used.

### Molecular biology

For all transgenic constructs, the desired sequences were amplified from appropriate cDNA sources (that is, antennal cDNA from *D. melanogaster*, *A. mellifera* or *A. polyphemus*, or *M. musculus* cDNA) using the KAPA HiFi PCR kit (Kapa Biosystems). PCR products were T:A cloned into pGEM-T Easy (Promega), sequenced and subcloned with restriction enzymes, whose recognition sites were incorporated in the PCR primers (Supplementary Table 2), into the pUAST-attB vector^29^ or, for C-terminal tagging, a pUAST-EGFP-attB vector. Deletions and point mutations in the SNMP1-coding sequence (Supplementary Fig. 1) were introduced by PCR-based deletion and site-directed mutagenesis of pGEM-T SNMP1, respectively (Supplementary Table 2). For the pUAST-SNMP1/NINAD:EGFP construct, PCR stitching was used to generate a sequence encoding an EGFP-tagged chimeric protein comprising the SNMP1 ectodomain (residues 28–450) flanked by the N- and C-terminal intracellular/transmembrane domains of NINAD (residues 1–33 and 448–513, respectively). For HEK 293 cell expression, SNMP1:EGFP was cloned in a modified version of pCG^60^. For *Xenopus* oocyte expression, SNMP1:EGFP was cloned in pXpress^51^ to generate the desired capped RNA (cRNA). All the plasmids were fully sequence-verified.

### Biochemistry

Antennal protein extracts from ∼500 third antennal segments of wild-type flies, disrupted in a TissueLyzer (Qiagen), were made by incubating lysed antennae in 250 ml of extraction buffer (20 mM Tris (pH 7.5), 100 mM NaCl, 5 mM KCL, 1.5 mM MgCl2, 4% glycerol, 0.02% *n*-dodecyl-D-maltoside) for 90 min at 4 °C, followed by centrifugation at 12,000*g* for 15 min at 4 °C (ref. 34). To analyse the presence of disulfide bonds in SNMP1, protein extracts were incubated in the presence or absence of either 100 mM dithiothreitol (Merck) or 10% β-mercaptoethanol (Promega) during 3 min at 95 °C. To analyse the N-glycosylation status of SNMP1, protein extracts were treated with PNGase F (New England Biolabs) in the absence or presence of 10% SDS during 1 h at 37 °C. After these treatments, extracts were separated on 4–20% precast gels (NuSep) and transferred to Hybond-ECL membrane (Amersham), which were probed with primary antibodies against SNMP1 (ref. 16) diluted to 1:1,000. Goat α-IgG rabbit secondary antibodies coupled to horseradish peroxidase (Promega W4011) were diluted 1:10,000. Blots were developed with medical X-ray films (Fujifilm) using the ECL Plus Western blotting detection system (GE Healthcare). The resulting films were scanned without any automatic gain.

### Cell culture and transfection

HEK 293 cells (American Type Culture Collection, LGC Standards; not authenticated or tested for mycoplasma) were maintained in Dulbecco's modified Eagle's medium (GIBCO) supplemented with 10% fetal bovine serum, without antibiotics. To establish a stable HEK 293 cell line expressing EGFP-tagged SNMP1, HEK 293 cells were grown to 50–60% confluence in 10-cm dishes and transfected with 1 μg of pCG-SNMP1:EGFP using Lipofectamine 2000 (Life Technologies). Cells were split after 24 h and kept under G418 selection (500 μg ml^−1^) for 21 days. Individual clones were then expanded and tested for SNMP1:EGFP expression.

### Histology

For immunofluorescence analysis^61^,^62^, 14-μm antennal cryosections in Tissue-Tek optimum cutting temperature (O.C.T.) compound (Sakura) were collected on slides and fixed for 7 min in 4% formaldehyde in phosphate-buffered saline (PBS). After washing twice for 10 min in PBS, sections were permeabilized for 30 min in PBS+0.1% Triton X-100 (P/T) and blocked in 5% heat-inactivated normal goat serum in P/T (P/T/S) for 30 min. Primary antibodies were diluted in P/T/S and applied to slides placed horizontally in humidified chambers and left for 36 h at 4 °C. After washing for three times for 10 min in P/T, slides were blocked again in P/T/S for 30 min and incubated with secondary antibodies diluted in P/T/S in humidified chambers in the dark for 2 h. Slides were then washed three times for 5 min in P/T and mounted in Vectashield (Vector Labs). The primary antibodies used were rabbit α-SNMP1 (C terminus)^62^ (diluted 1:200), rabbit α-GFP (Invitrogen A-6455) (1:1,000) and guinea pig α-ORCO^34^ (1:1,000). Rabbit polyclonal antibodies against the SNMP1 ectodomain (used only in Fig. 3b) were raised against the synthetic peptide FDEWKDKYDLEDDVVEDTV and affinity purified (Proteintech Group, Inc (Chicago)) and diluted to 1:200. The secondary antibodies used were Alexa488-conjugated α-rabbit IgG (Invitrogen A11034) and Cy3-conjugated anti-guinea pig IgG (Jackson Immunoresearch 106-166-003) and were diluted to 1:1,000.

HEK 293 cells expressing SNMP1:EGFP were grown on glass coverslips and washed several times with Dulbecco's modified Eagle's medium before fixing them for 2 min with 4% paraformaldehyde in PBS. Cells were incubated for 5 min with PBS containing 2% BSA with or without 0.2% Triton X-100 (Sigma) for permeabilized and unpermeabilized conditions, respectively. Cells were then incubated during 1 h with rabbit polyclonal antibodies against GFP (Invitrogen A-6455) diluted to 1:1,000 in PBS with 2% BSA, washed five times with PBS and incubated for 1 h with Cy3-conjugated α-rabbit IgG (Jackson Immunoresearch 106-166-003) diluted to 1:1,000 in PBS with 2% BSA.

Microscopy was performed using a Zeiss LSM 510 Meta Upright Laser Scanning Confocal Microscope. Confocal images were processed with ImageJ^63^ and Adobe Photoshop CS4. Tissue orientation in all images is dorsal up/lateral left.

For quantification of dendrite immunofluorescence, SNMP1 signal was quantified selecting the area of a sensillar hair and obtaining the GFP Integrated Density values for the selected area and normalized with the corresponding ORCO signal in the same area to avoid the variation in staining intensity that arises from heterogeneous permeation of antibodies within the sensillar shaft. Image J was used to obtain the Integrated Density values. Data were analysed using Prism 6 software. Normality was assessed with D'Agostino–Pearson test, and the Kruskal–Wallis test was used to compare means among genotypes. The Dunn's test was used to correct the *P* values for multiple comparisons.

### Electrophysiology

Extracellular recordings of OSN activity in individual sensilla of 4- to 10-day-old flies were performed using standard methodology^30^,^34^. The sample sizes (*n*) indicated in the figures correspond to biological replicates (different sensilla), with a maximum of three sensilla per animal, mixed genders. Exact sample sizes for each experimental/group condition are provided in Supplementary Table 1. Genotypes (not blinded to experimenter) were interleaved to minimise effects of time-of-day.

For odour presentation in an airstream, 10 μl of odorant were added to a 6 × 7.5-mm absorbent strip (Sugi, Kettenbach), which was placed inside a 1-ml tuberculin syringe (Becton, Dickinson and Company). A charcoal-filtered airflow (35 ml s^−1^) was used to deliver odours to the preparation through a 10-ml serological pipette that was trimmed to remove the tapered tip, and the cut end positioned 15 mm away from the preparation. Half of this airflow was diverted through the odour syringe during odour stimulation periods (1 s) under the control of the Syntech CS-55 Stimulus controller. cVA (CAS No. 6186-98-7, Pherobank; purity 99%) and (*Z*)-11-hexadecenal (CAS No. 53939-28-9; Sigma-Aldrich) were diluted v/v in paraffin oil as indicated in the figures. For each recording session, we determined the time of onset of the response of a control sensillum to 10% cVA (usually ∼200 ms). Corrected responses were quantified by counting spikes in a 0.5-s window from this time point, subtracting the number of spikes in a 0.5 s window before stimulation, and doubling the result to obtain spikes/s.

For odour presentation at close range, 2 μl of odorant were added to the tip of a 1-mm filter paper (Whatman). Using a fine micromanipulator the filter paper tip was approached within ∼0.1 mm of the third antennal segment, avoiding direct contact^34^. The stimulus was presented once in a recording window of 13 s. The response was quantified similarly to that described above by counting spikes in 0.5-s windows before and after approach of the filter paper, avoiding the window immediately before the point of closest approach when increases in spike frequency were observed in the rescue animals.

Sample sizes were fixed before data analysis, based on preliminary studies. Data were analysed using Prism 6 software. Normality was assessed with D'Agostino–Pearson tests followed by Mann–Whitney, one-way analysis of variance or Kruskal–Wallis tests to compare means among genotypes as appropriate. The Dunn's test was used to correct the *P* values for multiple comparisons. Differences were considered significant if the adjusted *P* value was <0.05. Unless indicated otherwise in the figure legends, *post hoc* tests were performed to compare the neuronal responses conferred by the mutant SNMP1 proteins with those restored by full-length SNMP1.

### Single-molecule imaging

Imaging of SNMP1:EGFP in *Xenopus* oocyte membranes by total internal reflection fluorescence microscopy was performed by injecting cRNA encoding SNMP1:EGFP into *Xenopus* oocytes at a concentration of 0.02 μg μl^−1^ in a total of 50 nl water per cell. About 12–24 h after injection and expression at 15 °C, cells were enzymatically treated with hyaluronidase (1 mg ml^−1^, Sigma) and neuraminidase (1 U ml^−1^, Sigma) for 15 min at 4 °C and manually devitellinized. Multiple oocytes were placed on a coverslip and movies of 500 frames of an area of plasma membrane of 25.6 × 25.6 μm were taken at 30 frames per second with a back-illuminated EMCCD camera (Andor iXon DV-897 BV). EGFP was excited at 488 nm and measured using a 525/50 emission filter. To extract fluorescence intensities, we summed the pixel counts in defined regions of interest around the centre positions of the spots. Traces of fluorescence intensity were examined by eye for the presence of multiple bleaching steps.

### Surface plasmon resonance

The affinity of a series of ligands was measured using a CD36-modified SPR sensor^42^. The SPR sensor was constructed on a dove prism by depositing a 1-nm chromium adhesion layer and a 50-nm Au film. The Au film was modified with a 3-MPA-LHDLHD-OH self-assembled monolayer synthesized as described^42^. The SPR sensor and a fluid delivery system were inserted in a miniature SPR instrument^64^. The 3-MPA-LHDLHD-OH monolayer was further reacted in the SPR instrument to create a monolayer competent to bind His-tag proteins. The CD36 ectodomain (amino acids 30–439) was expressed in a pFastBac1 transfer plasmid with the viral sequence, an epitope FLAG and hexahistidine tag on the N terminus and immobilized on the SPR chip by exposing a 5-μg ml^−1^ His-tagged CD36 for 15 min. Aqueous solutions of the ligands were prepared in PBS buffer at different concentrations ranging from sub-μM to mM. Dimethylsulfoxide at 0.1% was added to PBS for hydrophobic ligands insoluble in pure PBS. The SPR experiment was recorded in real-time, and successive injections of the ligands at increasing concentrations established the affinity curve. A Langmuir isotherm was fitted to the affinity curves to extract the *K*_d_ and maximum SPR signal (Δ*λ*_SPR,max_) using MatLab curve-fitting tools. The ligands tested were the following: farnesol (CAS No. 4602-84-0; Aldrich); (*Z*)-11-hexadecenal (CAS No. 53939-28-9; Sigma-Aldrich); mixture of (+)-limonene and (−)-limonene (CAS No. 5989-27-5 and 5989-54-8, respectively; Sigma-Aldrich); bombykol (CAS No. 765-17-3; Pherobase); 11-*cis*-vaccenyl acetate (CAS No. 6186-98-7; Cayman Chemicals); ethyl acetate (CAS No. 141-78-6; Fluka); isoamyl acetate (CAS No. 123-92-2; Sigma-Aldrich); hexyl acetate (CAS No. 142-92-7; Fluka); and ethyl butyrate (CAS No. 105-54-4; Aldrich). We note that cVA from Pherobank showed affinity for CD36 only at the highest ligand concentration (>32 μM) in this assay; the discrepancy between the binding ability of these different sources of pheromone is unknown.

### Homology modelling and tunnel analysis

The secondary structure alignment of *Drosophila* SNMP1, and human CD36, SR-BI and LIMP-2 (Supplementary Fig. 7) was generated with PROMALS3D^65^. Structural models of the ectodomain of SNMP1 (residues 46–449) were built using Modeller (mod9.12)^66^ based on the chain A of the crystal structure of *Homo sapiens* LIMP-2 37–429 (PDB ID: 4F7B^5^; the other published LIMP-2 structures are very similar^45^). The only secondary structure constraint imposed is the bond between residues C265 and C330 since this bond is not conserved between the template and the target. The models generated using standard Modeller energy functions (molpdf and DOPE) were highly similar; the illustrated model was chosen based on accessibility of putatively glycosylated Asn residues (N66, N213, N226 and N440). These Asn residues were modified with fucosylated glycans using GLYCAM Web (Woods Group (2005–2014) GLYCAM Web. Complex Carbohydrate Research Center, University of Georgia, Athens, GA; www.glycam.com), using the default parameter file Glycam06. Tunnels were predicted within the ectodomain with Caver Analyst 1.0 (ref. 47) (www.caver.cz), using the default minimal probe radius of 0.9 Å. We found five tunnels to describe the cavity observed in the full-length SNMP1. Within residues lining these tunnels 12 were defined as bottlenecks: F56; F60; F115; M222; T274; T290; D292; P398; F399; K403; L405; and L439. Two of these, T274 and L439, located near the exit of the tunnel (close to the membrane), as well as V353, near the entrance, were chosen for mutagenesis. As a control, we also mutated A401, which lies within the tunnel but is not predicted to be a bottleneck. Transmembrane domains of SNMP1 were built using Pymol (The PyMOL Molecular Graphics System, Version 1.5.0.4 Schrödinger, LLC), based on secondary structure predictions from PSIPRED^67^. Model images were generated with VMD (www.ks.uiuc.edu/Research/vmd) (developed with NIH support by the Theoretical and Computational Biophysics group at the Beckman Institute, University of Illinois at Urbana-Champaign). The representation in Fig. 8a was rendered with VMD and POV-Ray, and PDB2PQR was used to highlight surface charges. Model coordinates are provided in Supplementary Data 1.

### Data availability

All relevant data supporting the findings of this study are available from the corresponding author on request.

## Additional information

**How to cite this article:** Gomez-Diaz, C. *et al.* A CD36 ectodomain mediates insect pheromone detection via a putative tunnelling mechanism. *Nat. Commun.* 7:11866 doi: 10.1038/ncomms11866 (2016).

## Supplementary Material

Supplementary Information Supplementary Figures 1-7 and Supplementary Tables 1-2

Supplementary Data 1 SNMP1 ectodomain protein homology model
