# Supplementary material for: A CD36 ectodomain mediates insect pheromone detection via a putative tunnelling mechanism
Source: Nat Commun. 2016 Jun 15;7:11866. doi: 10.1038/ncomms11866 (PMC4912623; doi:10.1038/ncomms11866)
Supplement: Supplementary Information — Supplementary Figures 1-7 and Supplementary Tables 1-2 [file ncomms11866-s2.pdf]

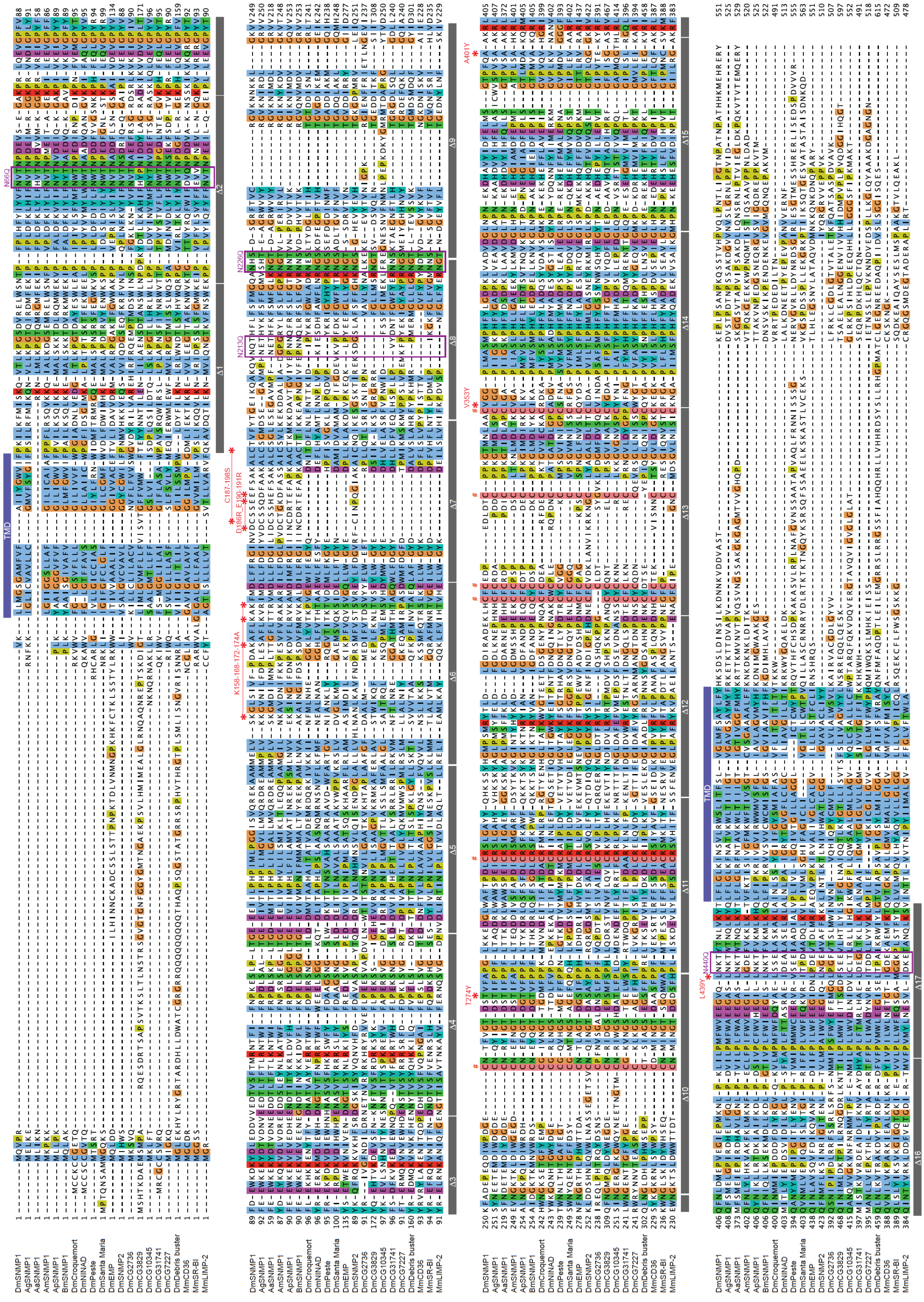

Supplementary Figure 1 | Multiple sequence alignment of CD36 family proteins.

Annotated alignment of the amino acid sequences of various insect SNMP1 proteins (Dm = *Drosophila melanogaster*; Ag = *Anopheles gambiae*; Aa = *Aedes aegypti*; Am = *Apis mellifera*; Ap = *Antheraea polyphemus*; Bm = *Bombyx mori*), twelve other *Drosophila melanogaster* CD36-related proteins, and the three mouse (Mm = *Mus musculus*) CD36 family members. Transmembrane domains (TMD) (predicted using TOPCONS; topcons.cbr.su.se*)* are marked with blue bars and conserved disulfide bond-forming cysteines are labeled with orange hash marks. Predicted SNMP1 N-glycosylation sites (NXS/T) (using NetNGlyc 1.0; www.cbs.dtu.dk/services/NetNGlyc) are highlighted with purple boxes. The grey bars beneath the alignment indicate the positions of the scanning series of 25-amino acid deletions in the SNMP1 ectodomain (Fig. 3e-f). Other SNMP1 residues mutated in this study are labeled with red asterisks; those linked by a line indicate that these sites were mutated within a single transgene.

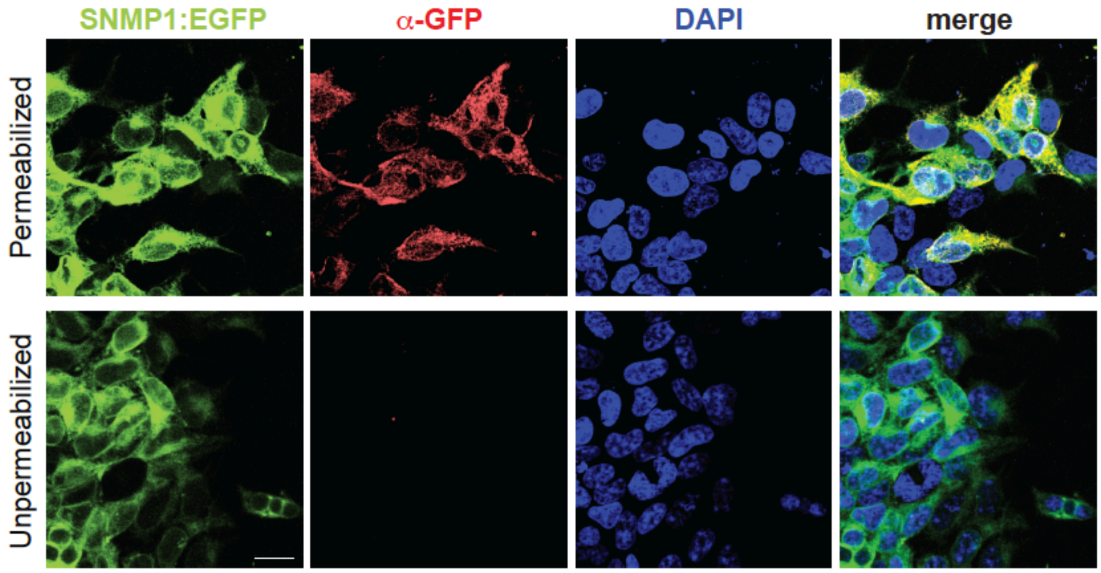

Supplementary Figure 2 | Membrane topology of SNMP1.
Native EGFP fluorescence (green) and α-GFP immunofluorescence (red) of HEK 293 cells expressing SNMP1:EGFP, under membrane permeabilized and unpermeabilized conditions. The DAPI counterstain (blue) labels cell nuclei. The scale bar represents 20 μm.

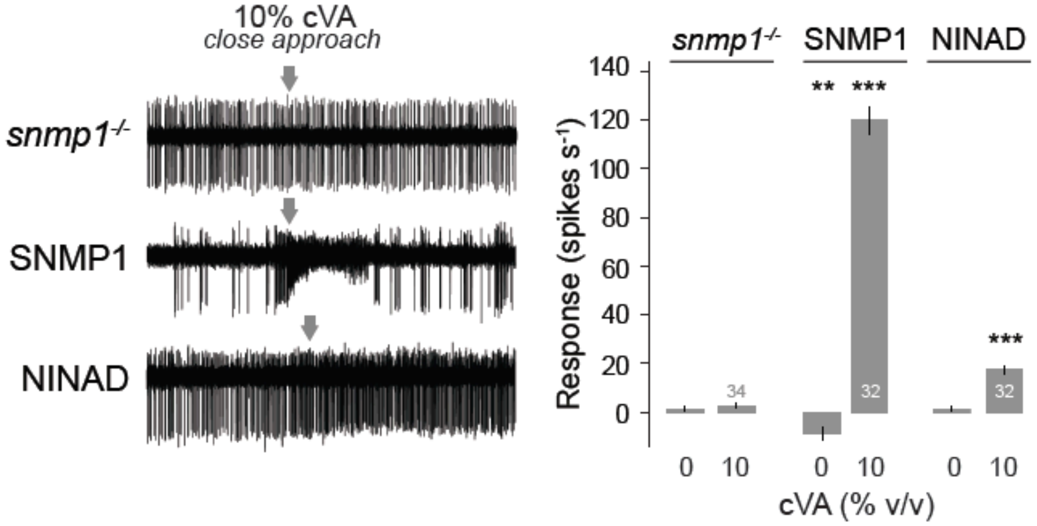

Supplementary Figure 3 | NINAD weakly restores cVA responses.
Left: Representative traces of electrophysiological recordings of OR67d neurons in a close-range stimulation assay in *snmp1* homozygous mutant, SNMP1 rescue and NINAD rescue flies stimulated with 10% cVA. The grey arrows indicate the approximate time of closest stimulus position to the sensillum; increases in spike frequency were observed in the rescue animals as the stimulus was approaching this point. Right: Mean neuronal responses ±SEM to the indicated stimuli in each genotype. The *n* is shown in grey or white above or within the 10% cVA stimulus bar. There are significant statistical differences in neuronal responses due to genotype (Kruskal-Wallis, p<0.0001). Significant differences in cVA responses of the different genotypes compared to *snmp1*$^{-/-}$ are indicated (** p<0.01, *** p<0.001).

a

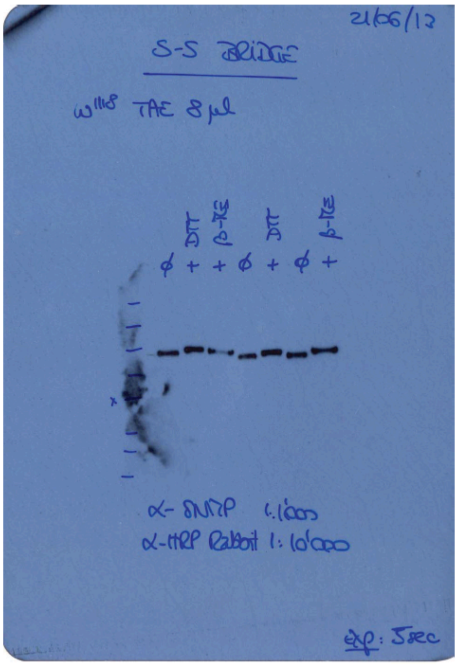

b

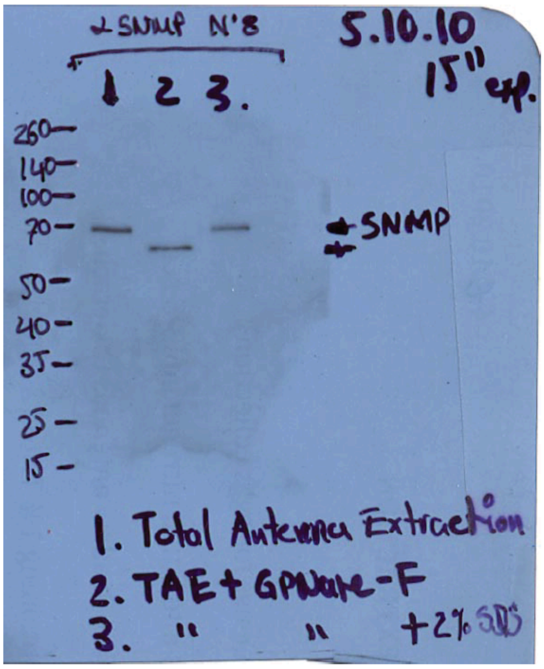

Supplementary Figure 4 | Analysis of the presence of intramolecular disulfide bonds and N-glycosylation in SNMP1.
(a) Complete film of the experiment shown in Fig. 4a.
(b) Complete film of the experiment shown in Fig. 5a.

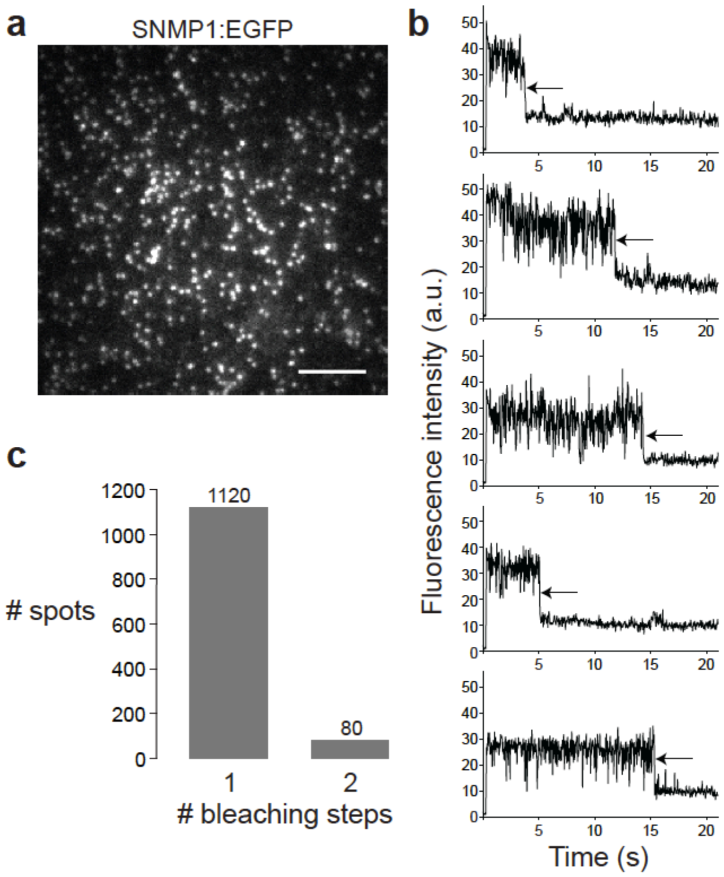

Supplementary Figure 5 | SNMP1:EGFP is principally monomeric.
(a) Single molecule fluorescence localization in membranes of live *Xenopus* oocytes visualized with total internal reflection fluorescence microscopy. Molecules of SNMP1:EGFP were detected as bright spots. The scale bar represents 5 µm.
(b) Representative examples of traces of emitted fluorescence (arbitrary units) from individual spots of oocytes expressing SNMP1:EGFP, revealing single prominent bleaching steps (arrows).
(c) Quantification of observed bleaching steps of EGFP signals in oocytes expressing SNMP1:EGFP.

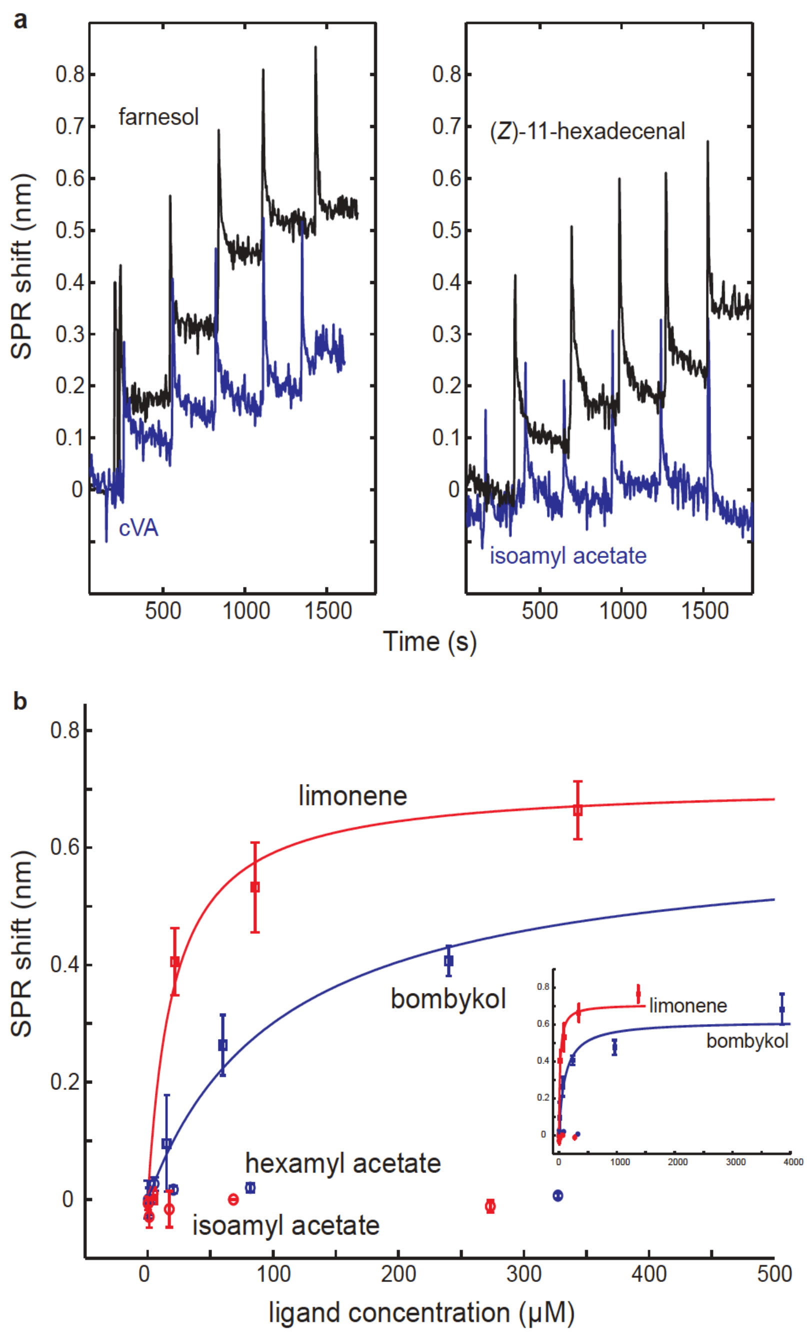

Supplementary Figure 6 | SPR sensorgram for binding of ligands.
(a) SPR sensorgram for binding of farnesol (concentrations: 0.29, 1.15, 4.59, 18.4 and 73.4 μM) and cVA (concentrations: 0.33, 1.31, 5.23, 20.9 and 83.7 μM) (left) and (*Z*)-11-hexadecenal (concentrations: 0.18, 0.73, 2.93, 11.7, and 47.0 μM) and isoamyl acetate (concentrations: 0.32, 1.28, 5.11, 20.5, 81.8 and 327 μM) (right). The CD36 sensor chips were exposed to increasing concentrations of the ligands and the equilibrium response was measured for the dose-response curves. The spikes in each trace coincide with the injection of successive stimulus doses.

(b) Concentration-response curves of $\Delta\lambda_{SPR}$ for the indicated ligands, which were used to calculate the corresponding dissociation constants (K_d). The inset represents the same data on an extended x-axis to show the full curves for limonene and bombykol.

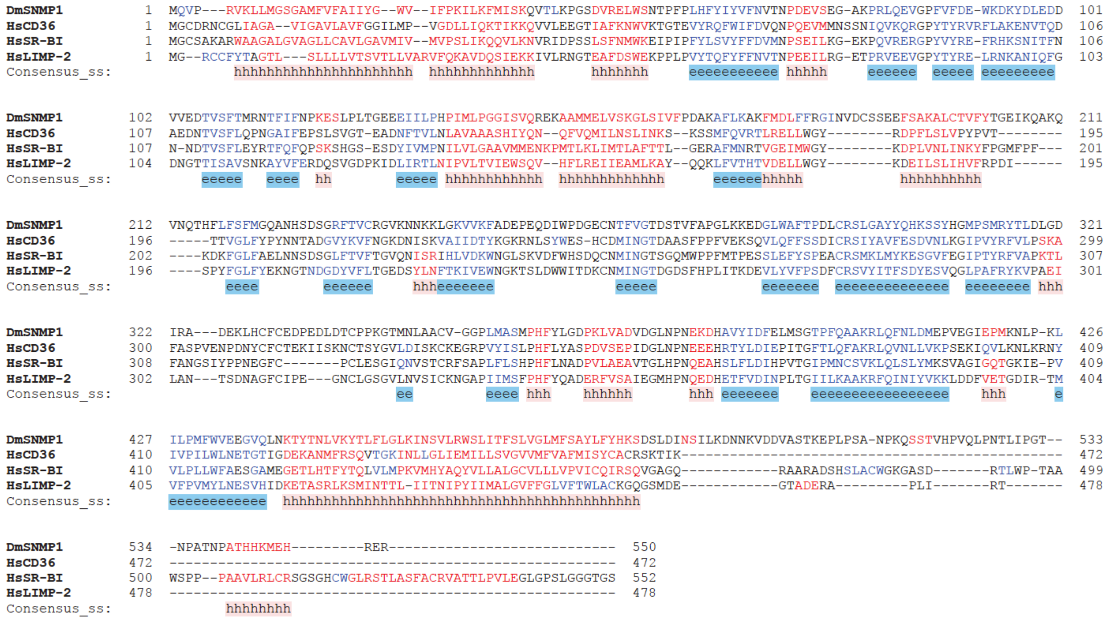

Supplementary Figure 7 | Secondary structure comparison of CD36 proteins.

Multiple sequence alignment of *Drosophila melanogaster* (Dm) SNMP1, and *Homo sapiens* (Hs) CD36, SR-BI and LIMP-2, colored for predicted secondary structural features (red: α-helix; blue: β-strand). The consensus predicted secondary structure (Consensus_ss) is shown below the aligned sequences.

Supplementary Table 1 | Electrophysiological response data.

Strain	Stimulus	Mean	± SEM	*n*
wildtype	solvent	-2.1	2.4	24
	1% cVA	32.4	5.1	24
	10% cVA	76.1	6.3	24
snmp1$^{-/-}$	solvent	-0.1	1.6	14
	1% cVA	-1.4	2.5	14
	10% cVA	1.1	1.2	14
SNMP1	solvent	1.8	0.9	25
	1% cVA	52.1	4.0	25
	10% cVA	80.6	4.2	25
CD36	solvent	0.0	0.6	18
	1% cVA	2.0	1.0	18
	10% cVA	14.4	2.4	18
SNMP1:EGFP	solvent	1.3	1.4	28
	1% cVA	59.5	3.9	28
	10% cVA	88.1	4.4	28
*Apol*SNMP1:EGFP	solvent	-0.2	2.0	17
	1% cVA	20.0	5.2	17
	10% cVA	43.1	5.5	17
*Amel*SNMP1:EGFP	solvent	-0.2	0.3	24
	1% cVA	19.1	3.1	24
	10% cVA	47.1	2.5	24
HR13	solvent	0.4	1.3	9
	1% hex	-0.7	2.2	9
	10% hex	5.5	1.5	9
HR13 + CD36	solvent	-1.1	1.5	14
	1% hex	13.7	2.1	14
	10% hex	32.3	4.4	14
HR13 + SNMP1	solvent	2.8	1.1	15
	1% hex	49.0	3.8	15
	10% hex	97.2	7.0	15
SNMP1$^{\Delta N}$	solvent	3.0	1.0	23
	1% cVA	43.3	4.5	23
	10% cVA	73.6	5.7	23
SNMP1$^{\Delta C}$	solvent	0.2	1.2	18
	1% cVA	31.8	3.5	18
	10% cVA	54.7	4.5	18
NINAD	solvent	1.0	0.8	25
	1% cVA	0.9	0.8	25
	10% cVA	0.2	0.9	25
SNMP1/NINAD:EGFP	solvent	0.1	1.2	23
	1% cVA	23.1	3.1	23
	10% cVA	52.9	5.4	23
SNMP1^{C265S}	solvent	-2.4	1.2	10
	1% cVA	-2.6	2.0	10
	10% cVA	-3.0	1.3	10
SNMP1^{C294S}	solvent	-1.6	1.2	16
	1% cVA	-2.6	1.0	16
	10% cVA	-1.2	1.1	16
SNMP1^{C332S}	solvent	-4.3	2.2	12
	1% cVA	-4.2	1.5	12
	10% cVA	-3.2	1.9	12
SNMP1$^{C330-332-341-352S}$	solvent	-3.1	1.9	9
	1% cVA	-2.1	1.2	9
	10% cVA	-5.6	1.1	9
SNMP1	solvent	-2.3	1.5	12
	1% cVA	56.0	7.2	12
	10% cVA	94.3	4.5	12
SNMP1^{N66Q}	solvent	-1.6	0.9	9
	1% cVA	-2.8	0.9	9
	10% cVA	-6.0	2.7	9
SNMP1^{N213Q}	solvent	1.7	3.5	12
	1% cVA	38.2	2.9	12
	10% cVA	81.5	5.1	12
SNMP1^{N226Q}	solvent	0.2	1.3	13
	1% cVA	30.2	4.7	13
	10% cVA	57.4	6.9	13

SNMP1^{N440Q}	solvent	1.7	2.1	12
	1% cVA	41.6	6.3	12
	10% cVA	91.3	10.5	12
SNMP1$^{N213-440Q}$	solvent	0.6	1.2	21
	1% cVA	25.7	3.8	21
	10% cVA	52.4	3.2	21
SNMP1$^{N213-226-440Q}$	solvent	2.0	1.0	17
	1% cVA	2.4	1.6	17
	10% cVA	0.0	1.4	17
SNMP1	solvent	0.0	0.8	10
	1% cVA	67.2	6.5	10
	10% cVA	92.2	5.8	10
SNMP1$^{K158-168-172-174A}$	solvent	3.2	2.3	24
	1% cVA	55.8	5.4	24
	10% cVA	92.0	6.3	24
SNMP1$^{D186R_E190-191R}$	solvent	-0.1	1.4	19
	1% cVA	3.2	2.3	19
	10% cVA	14.5	2.6	19
SNMP1$^{C187-198S}$	solvent	0.9	1.3	20
	1% cVA	6.8	2.1	20
	10% cVA	24.9	3.5	20
SNMP1	solvent	1.0	1.6	27
	0.01% cVA	3.5	2.1	27
	0.0316% cVA	3.8	2.2	27
	0.1% cVA	7.1	2.3	27
	0.316% cVA	23.4	2.9	27
	1% cVA	39.5	3.0	27
	3.16% cVA	60.67	3.5	27
	10% cVA	81.6	5.6	27
	31.6% cVA	95.5	5.4	27
SNMP1^{T274Y_L439Y}	solvent	0.1	1.0	30
	0.01% cVA	2.4	2.0	30
	0.0316% cVA	-0.8	1.5	30
	0.1% cVA	4.1	1.7	30
	0.316% cVA	6.1	1.9	30
	1% cVA	13.0	2.0	30
	3.16% cVA	30.2	3.0	30
	10% cVA	44.3	3.2	30
	31.6% cVA	54.9	4.2	30
SNMP1^{A401Y}	solvent	-2.2	1.6	27
	0.01% cVA	3.8	2.7	27
	0.0316% cVA	4.4	2.2	27
	0.1% cVA	7.3	2.1	27
	0.316% cVA	26.1	3.0	27
	1% cVA	36.0	3.2	27
	3.16% cVA	48.7	4.3	27
	10% cVA	67.4	5.0	27
	31.6% cVA	86.9	6.0	27
SNMP1^{V353Y}	solvent	2.9	2.4	16
	0.01% cVA	-0.4	2.4	16
	0.0316% cVA	7.1	2.2	16
	0.1% cVA	-2.9	2.8	16
	0.316% cVA	16.4	3.2	16
	1% cVA	29.1	2.6	16
	3.16% cVA	52.0	4.6	16
	10% cVA	61.0	4.8	16
	31.6% cVA	83.1	6.2	16
HR13 + SNMP1	solvent	2.8	1.1	15
	0.1% hex	13.2	2.8	15
	1% hex	49.0	3.8	15
	10% hex	97.2	7.0	15
HR13 + SNMP1^{T274Y_L439Y}	solvent	2.0	2.4	11
	0.1% hex	-0.9	2.2	11
	1% hex	15.1	3.3	11
	10% hex	45.3	8.0	11
HR13 + SNMP1^{A401Y}	solvent	4.6	2.0	11
	0.1% hex	13.8	2.0	10
	1% hex	57.8	6.2	10
	10% hex	113.3	8.2	10
HR13 + SNMP1^{V353Y}	solvent	4.0	1.4	15
	0.1% hex	11.7	1.5	15

	1% hex	32.4	3.0	15
	10% hex	81.5	5.1	15
snmp1$^{-/-}$ (close-range)	solvent	1.4	1.1	34
	10% cVA	2.6	1.0	34
SNMP1 (close-range)	solvent	-8.9	2.4	32
	10% cVA	119.9	5.5	32
CD36 (close-range)	solvent	0.46	1.5	35
	10% cVA	39.8	4.2	35
NINAD (close-range)	solvent	1.7	1.1	32
	10% cVA	17.9	1.7	32

Supplementary Table 2 | Oligonucleotides for transgene generation.

Transgene	Oligonucleotide sequence 5'⇒3'
DmSNMP1 sense	CAATTGCACACCATGCAAGTACCTCGGGTTAAG
DmSNMP1 antisense	CTCGAGTTAGTAGCGCTCCCGATGCTC
AmSNMP1 sense	GCGGCCGCCACACCATGAAGCCGAAAAAATTAGG
AmSNMP1 antisense (no stop codon)	GTCGACATCCGCGTCGAGATTAGGGG
ApolSNMP1 sense	GCGGCCGCCACACCATGTTATTGCCAAAGCCGTTG
ApolSNMP1 antisense (no stop codon)	CTCGAGCATGGTCACTTTAGGTGGTTC
MmCD36 sense	CAATTGCACACCATGGGCTGTGATCGGAACT
MmCD36 antisense	CTCGAGTTATTTTCCATTCTTGGATTTGC
MmCD36 antisense (no stop codon)	CTCGAGTTTTCCATTCTTGGATTTGC
DmSNMP1$^{\Delta N\text{-term}}$ sense	CAATTGCACACCATGAAGCTGCTCATGGGGTCGGG
DmSNMP1$^{\Delta C\text{-term}}$ antisense	CTCGAGTTATTTGTGGTAGAAAAGATAGG
NINAD sense	CAATTGCACACCATGTGCTGCAGCTGCTGTGGGGTGA
NINAD antisense	CTCGAGTTAAAAGTTTCGTTCGACCACGTTG
NINAD antisense (no stop codon)	CTCGAGAAAGTTTCGTTCGACCACGTTG
DmSNMP1:NinaD sense	TTAGTGAAATACACACTGAATCTACCGGCT
DmSNMP1:NinaD antisense (no stop codon)	CTTGAGAATTTTGGGCCAGCATATTATGGC
DmSNMP1$^{\Delta 1}$ sense	TGGCTGGGTTATCTTTAATACTCCATTCCCAC
DmSNMP1$^{\Delta 1}$ antisense	GTGGGAATGGAGTATTAAAGATAACCCAGCCA
DmSNMP1$^{\Delta 2}$ sense	TCGGGAACTCTGGTCCAAGCCACGGCTACAGG
DmSNMP1$^{\Delta 2}$ antisense	CCTGTAGCCGTGGCTTGGACCAGAGCACACCTTCCCGA
DmSNMP1$^{\Delta 3}$ sense	GGTATCCGAAGGAGCTGTAGAGGACACGGTCA
DmSNMP1$^{\Delta 3}$ antisense	TGACCGTGTCCTCTACAGCTCCTTCGGATACC
DmSNMP1$^{\Delta 4}$ sense	TTTGGAGGATGATGTCGAGGAGGAGATTATTT
DmSNMP1$^{\Delta 4}$ antisense	AAATAATCTCCTCCTCGACATCATCCTCCAAA
DmSNMP1$^{\Delta 5}$ sense	GCTGCCACTTACGGGCATGGAGCTGGTTTCCA
DmSNMP1$^{\Delta 5}$ antisense	TGGAAACCAGCTCCATGCCCGTAAGTGGCAGC
DmSNMP1$^{\Delta 6}$ sense	GGAAAAGGCTGCCATGTTATTTTTCCGCGGCA
DmSNMP1$^{\Delta 6}$ antisense	TGCCGCGGAAAAATAACATGGCAGCCTTTTCC
DmSNMP1$^{\Delta 7}$ sense	AGCTAAATTTATGGATACGGGAGAAATTAAGC
DmSNMP1$^{\Delta 7}$ antisense	GCTTAATTTCTCCCGTATCCATAAATTTAGCT
DmSNMP1$^{\Delta 8}$ sense	CTGCACCGTGTTTTACTCGGATTCTGGCAGAT
DmSNMP1$^{\Delta 8}$ antisense	ATCTGCCAGAATCCGAGTAAAACACGGTGCAG
DmSNMP1$^{\Delta 9}$ sense	GGGTCAGGCCAATCACGACGAACCGGAGCAAG
DmSNMP1$^{\Delta 9}$ antisense	CTTGCTCCGGTTCGTCGTGATTGGCCTGACCC
DmSNMP1$^{\Delta 10}$ sense	GGTAGTGAAATTCGCCCCGGGCTTGAAAAAGG
DmSNMP1$^{\Delta 10}$ antisense	CCTTTTTCAAGCCCGGGGCGAATTTCACTACC
DmSNMP1$^{\Delta 11}$ sense	TTCTACGGTCTTTGCACACAAGTCATCCTACC
DmSNMP1$^{\Delta 11}$ antisense	GGTAGGATGACTTGTGTGCAAAGACCGTAGAA
DmSNMP1$^{\Delta 12}$ sense	GGGCGCTTATTATCAGCTTCATTGTTTCTGCG
DmSNMP1$^{\Delta 12}$ antisense	CGCAGAAACAATGAAGCTGATAATAAGCGCCC
DmSNMP1$^{\Delta 13}$ sense	ACGTGCCGATGAGAAGGTTGGTGGTCCATTGA
DmSNMP1$^{\Delta 13}$ antisense	TCAATGGACCACCAACCTTCTCATCGGCACGT
DmSNMP1$^{\Delta 14}$ sense	GAATCTGGCGGCCTGTCTCAATCCGAATGAAA
DmSNMP1$^{\Delta 14}$ antisense	TTTCATTCGGATTGAGACAGGCCGCCAGATTC
DmSNMP1$^{\Delta 15}$ sense	TGCCGACGTTGATGGTAAGCGTTTGCAGTTTA

DmSNMP1$^{\Delta15}$ antisense	TAAACTGCAAACGCTTACCATCAACGTCGGCA
DmSNMP1$^{\Delta16}$ sense	ACCTTTTCAGGCTGCCTTGCCCATGTTTTGGG
DmSNMP1$^{\Delta16}$ antisense	CCCAAAACATGGGCAAGGCAGCCTGAAAAGGT
DmSNMP1$^{\Delta17}$ sense	TCTACCCAAGCTGATACTATTTCTGGGCCTAA
DmSNMP1$^{\Delta17}$ antisense	TTAGGCCCAGAAATAGTATCAGCTTGGGTAGA
DmSNMP1^{C265S} sense	CCAGATGGCGAGAGCAACACCTTTG
DmSNMP1^{C265S} antisense	CAAAGGTGTTGCTCTCGCCATCTGG
DmSNMP1^{C294S} sense	GCATTCACGCCGGATTTGAGCAGATCCCTGGGCGCTTAT
DmSNMP1^{C294S} antisense	ATAAGCGCCCAGGGATCTGCTCAAATCCGGCGTGAATG
DmSNMP1^{C332S} sense	CTTCATTGTTTCAGCGAAGATCCCG
DmSNMP1^{C332S} antisense	CGGGATCTTCGCTGAAACAATGAAG
DmSNMP1$^{C330\text{-}332S}$ sense	GATGAGAAGCTTCATAGCTTCAGCGAAGATCC
DmSNMP1$^{C330\text{-}332S}$ antisense	GGATCTTCGCAGAAGCTATGATGCTTCTCATC
DmSNMP1^{C341S} sense	ATCCCGAAGATCTGGACACGAGCCCGCCCAAGGGCACT
DmSNMP1^{C341S} antisense	TCATAGTGCCCTTGGGCGGGCTCGTGTCCAGATCTTCG
DmSNMP1^{C352S} sense	CTATGAATCTGGCGGCCAGCGTTGGTGGTCCATTGATG
DmSNMP1^{C352S} antisense	CATCAATGGACCACCAACGCTGGCCGCCAGATTCATAG
DmSNMP1^{N66Q} sense	CTATATCTACGTTTTCCAGGTAACCAATCCGGATG
DmSNMP1^{N66Q} antisense	CATCCGGATTGGTTACCTGGAAAACGTAGATATAG
DmSNMP1^{N213Q} sense	CAGGCCAAACAAGTCCAGCAAACTCACTTTTTG
DmSNMP1^{N213Q} antisense	CAAAAAGTGAGTTTGCTGGACTTGTTTGGCCTG
DmSNMP1^{N226Q} sense	GTTTATGGGTCAGGCCCAGCACTCGGATTCTGGC
DmSNMP1^{N226Q} antisense	GCCAGAATCCGAGTGCTGGGCCTGACCCATAAAC
DmSNMP1^{N440Q} sense	GAGGGCGTGCAGTTGCAGAAGACCTACACGAAC
DmSNMP1^{N440Q} antisense	GTTCGTGTAGGTCTTCTGCAACTGCACGCCCTC
DmSNMP1^{K158A} sense	GATGGAGCTGGTTTCCGCAGGATTAAGCATTGTC
DmSNMP1^{K158A} antisense	GACAATGCTTAATCCTGCGGAAACCAGCTCCATC
DmSNMP1$^{K168\text{-}172\text{-}174A}$ sense	CGGATGCCGCCGCCTTTTTGGCCGCTGCCTTTATGGATTTATTTTTCCGC
DmSNMP1$^{K168\text{-}172\text{-}174A}$ antisense	GCGGAAAAATAAATCCATAAAGGCAGCGGCCAAAAAGGCGGCGGCATCCG
DmSNMP1$^{D186R_E190\text{-}191R}$ sense	GCGGCATCAACGTACGTTGCTCCTCGAGAAGATTCTCCGCAAAGGCGCTC
DmSNMP1$^{D186R_E190\text{-}191R}$ antisense	GAGCGCCTTTGCGGAGAATCTTCTCGAGGAGCAACGTACGTTGATGCCGC
DmSNMP1$^{C187\text{-}198S}$ sense	GCATCAACGTAGATTCCTCCTCGGAAGAATTCTCCGCAAAGGCGCTCTCCACCGTGTTTTACACG
DmSNMP1$^{C187\text{-}198S}$ antisense	CGTGTAAAACACGGTGGAGAGCGCCTTTGCGGAGAATTCTTCCGAGGAGGAATCTACGTTGATGC
DmSNMP1^{T274Y} sense	GTGGGCACTGATTCTTACGTCTTTGCACCGGGC
DmSNMP1^{T274Y} antisense	GCCCGGTGCAAAGACGTAAGAATCAGTGCCCAC
DmSNMP1^{L439Y} sense	GGAGGGCGTGCAGTACAACAAGACCTACAC
DmSNMP1^{L439Y} antisense	GTGTAGGTCTTGTTGTACTGCACGCCCTCC
DmSNMP1^{A401Y} sense	CTGGAACACCTTTTCAGTACGCCAAGCGTTTGCAGT
DmSNMP1^{A401Y} antisense	ACTGCAAACGCTTGGCGTACTGAAAAGGTGTTCCAG
DmSNMP1^{V353Y} sense	AATCTGGCGGCCTGTTATGGTGGTCCATTGAT
DmSNMP1^{V353Y} antisense	ATCAATGGACCACCATAACAGGCCGCCAGATT
DmSNMP1 sense	CGTCTAGAATGCAAGTACCTCGGGTTAAG
DmSNMP1 antisense (no stop codon)	CGGGATCCGTAGCGCTCCCGATGCTCCAT
eGFP sense (for pCG)	GCGGCCGCCTACTTGTACAGCTCGTCCATGCCG
eGFP antisense (for pCG)	GGATCCCACACCATGAGCAAGGGCGAGGAGC
eGFP sense (for pXpress)	CTCGAGATGGTGAGCAAGGGCGAGGAG
eGFP antisense (for pXpress)	CTCGAGTTACTTGTACAGCTCGTCCAT

Restriction sites and/or *Drosophila* Kozak consensus sequence are included, where applicable, in the sequences (Dm = *Drosophila melanogaster*; Am = *Apis mellifera*; Ap = *Antheraea polyphemus;* Mm = *Mus musculus).*
